# Shrimp shapes a resistance trait against vibriosis by memorizing the colonization resistance of intestinal microbiota

**DOI:** 10.1371/journal.ppat.1012321

**Published:** 2024-07-11

**Authors:** Jianbo Yuan, Yang Yu, Shihao Li, Xiaojun Zhang, Chuntao Zhang, Roujing Li, Jie Hu, Shuqing Si, Chengyi Zhang, Jianhai Xiang, Fuhua Li

**Affiliations:** 1 CAS and Shandong Province Key Laboratory of Experimental Marine Biology, Center for Ocean Mega-Science, Institute of Oceanology, Chinese Academy of Sciences, Qingdao, China; 2 Key Laboratory of Breeding Biotechnology and Sustainable Aquaculture, Chinese Academy of Sciences, Wuhan, China; 3 University of Chinese Academy of Sciences, Beijing, China; Uppsala University, SWEDEN

## Abstract

Vibriosis is one of the most serious diseases that commonly occurs in aquatic animals, thus, shaping a steady inherited resistance trait in organisms has received the highest priority in aquaculture. Whereas, the mechanisms underlying the development of such a resistance trait are mostly elusive. In this study, we constructed vibriosis-resistant and susceptible families of the Pacific white shrimp *Litopenaeus vannamei* after four generations of artificial selection. Microbiome sequencing indicated that shrimp can successfully develop a colonization resistance trait against *Vibrio* infections. This trait was characterized by a microbial community structure with specific enrichment of a single probiotic species (namely *Shewanella algae*), and notably, its formation was inheritable and might be memorized by host epigenetic remodeling. Regardless of the infection status, a group of genes was specifically activated in the resistant family through disruption of complete methylation. Specifically, hypo-methylation and hyper-expression of genes related to lactate dehydrogenase (LDH) and iron homeostasis might provide rich sources of specific carbon (lactate) and ions for the colonization of *S*. *algae*, which directly results in the reduction of *Vibrio* load in shrimp. Lactate feeding increased the survival of shrimp, while knockdown of LDH gene decreased the survival when shrimp was infected by *Vibrio* pathogens. In addition, treatment of shrimp with the methyltransferase inhibitor 5-azacytidine resulted in upregulations of LDH and some protein processing genes, significant enrichment of *S*. *algae*, and simultaneous reduction of *Vibrio* in shrimp. Our results suggest that the colonization resistance can be memorized as epigenetic information by the host, which has played a pivotal role in vibriosis resistance. The findings of this study will aid in disease control and the selection of superior lines of shrimp with high disease resistance.

## Author summary

Vibriosis is a bacterial disease that commonly occurs in animals, and causes high mortality and severe economic loss in aquaculture every year. Therefore, shaping a steady inherited resistance trait in organisms has received the highest priority in artificial selection. Whereas, the mechanisms underlying the development of such a resistance trait are mostly elusive. In this study, we found that penaeid shrimp can adopt a defense mechanism of colonization resistance against vibriosis. Wherein, a specific enrichment of a single probiotic species (*Shewanella algae*) in the intestine plays pivotal roles in reducing the *Vibrio* load and controlling vibriosis. Further evidence revealed that this process might be memorized and regulated by the host’s epigenetic system. Activation of a subset of the host genes might provide proper niches for the enrichment of probiotics and simultaneous reduction of pathogens in the intestine through disruption of complete methylation. Together, our findings not only offer new insights into the key mechanisms in shaping disease resistance in shrimp, but also provide some clues for vibriosis control in aquaculture and for the selection of superior lines of shrimp with high disease resistance.

## Introduction

Vibriosis is one of the most common bacterial diseases that has been identified in most animals, including humans, fish, shrimp, and many other aquatic organisms, which generally causes high mortality and severe economic loss [1–4]. Owing to its economic and animal welfare importance, vibriosis resistance is arguably the most important target trait in the breeding goals of advanced aquaculture programs [5,6]. As the most widely cultured shrimp species, the Pacific white shrimp *Litopenaeus vannamei* has the highest value of all traded crustacean products [7]. However, shrimp farming continues to be seriously impaired by vibriosis, which is known as acute hepatopancreatic necrosis disease (AHPND) or early mortality syndrome (EMS) [8,9]. AHPND/EMS is caused by a *Vibrio* species, *Vibrio parahaemolyticus*, designated as *VP*_*AHPND*_ [10]. Since its first discovery in 2009, great efforts have been made to elucidate the pathology of AHPND/EMS and to control its outbreaks [11,12].

Screening and breeding of disease-resistant broodstock is regarded as an effective and sustainable approach for controlling the disease [13]. A number of disease-resistant families have been constructed in shrimp [14–17]. Based on these breeding lines, previous studies have mostly focused on identifying genetic variations and differentially expressed genes (DEGs) between resistant and susceptible families to seek targets that lead to disease resistance [15,18–22]. In our previous works, polymorphisms in *LvALF*, *TRAF6* and many other immune genes were suggested to be associated with disease resistance in shrimp [18,22]. However, the key mechanisms of disease resistance are still unclear, although many genetic clues have been provided.

In addition to the genetic basis, epigenetic remodeling likewise plays a functional role in shaping aquaculture traits [19]. Whereas, epigenetic studies in shrimp are still in their infancy. Environmental factors, including pathogens and artificial selection (a process by which humans choose individual organisms with certain phenotypic trait values for breeding), can exert influence on epigenetic changes to produce new phenotypes, which can be passed on to offspring [23]. DNA methylation is one of the most studied epigenetic mechanisms, which is primarily correlated with the down-regulations of gene expressions [19,24]. However, the functional role of methylation and other epigenetic regulators in shaping the aquaculture traits of shrimp has not been reported till present.

A dense and diverse microbial community inhabits the intestine and co-evolves with the host [25]. The intestinal microbiota is a pivotal and direct regulator in physiology, immunity, and health of organisms [26,27]. A major function of the intestinal microbiota is helping the host resist pathogen colonization and overgrowth of indigenous pathobionts, which is known as the defense mechanism of colonization resistance [28]. The intestines enriched with probiotics have been reported in some disease-resistant families of animals, which can effectively inhibit the growth of pathogens [26,29]. Therefore, the formation of a disease-resistant microbial community will have a profound impact on enhancing host resistance to pathogens. However, the intestinal microbiome composition is shaped by multiple factors [30]; it is not clear whether the host can construct a disease-resistant microbial community under artificial selection, and what can help its formation.

In this study, based on the resistant and susceptible families of *L*. *vannamei* obtained through selective breeding [15], we analyzed vibriosis resistance mechanisms in terms of both host and commensal microorganisms. The potential roles of epigenetic regulation and the intestinal microbiome in resistance to *Vibrio* were identified through methylome, transcriptome and microbiome sequencing and methyltransferase inhibitor treatment. To the best of our knowledge, the present study provides the first comprehensive investigation of whole-genome DNA methylation patterns and microbiome variations between the resistant and susceptible families. The final results suggested that there is a strong link between the hepatopancreas epigenetic system and the intestinal microbiome in forming a hepatopancreas-intestine barrier axis against *Vibrio*. This study offers novel insights into the role of epigenetic remodeling in colonization resistance.

## Results

### Differential performances between the resistant and susceptible shrimp families

**Fig 1:**
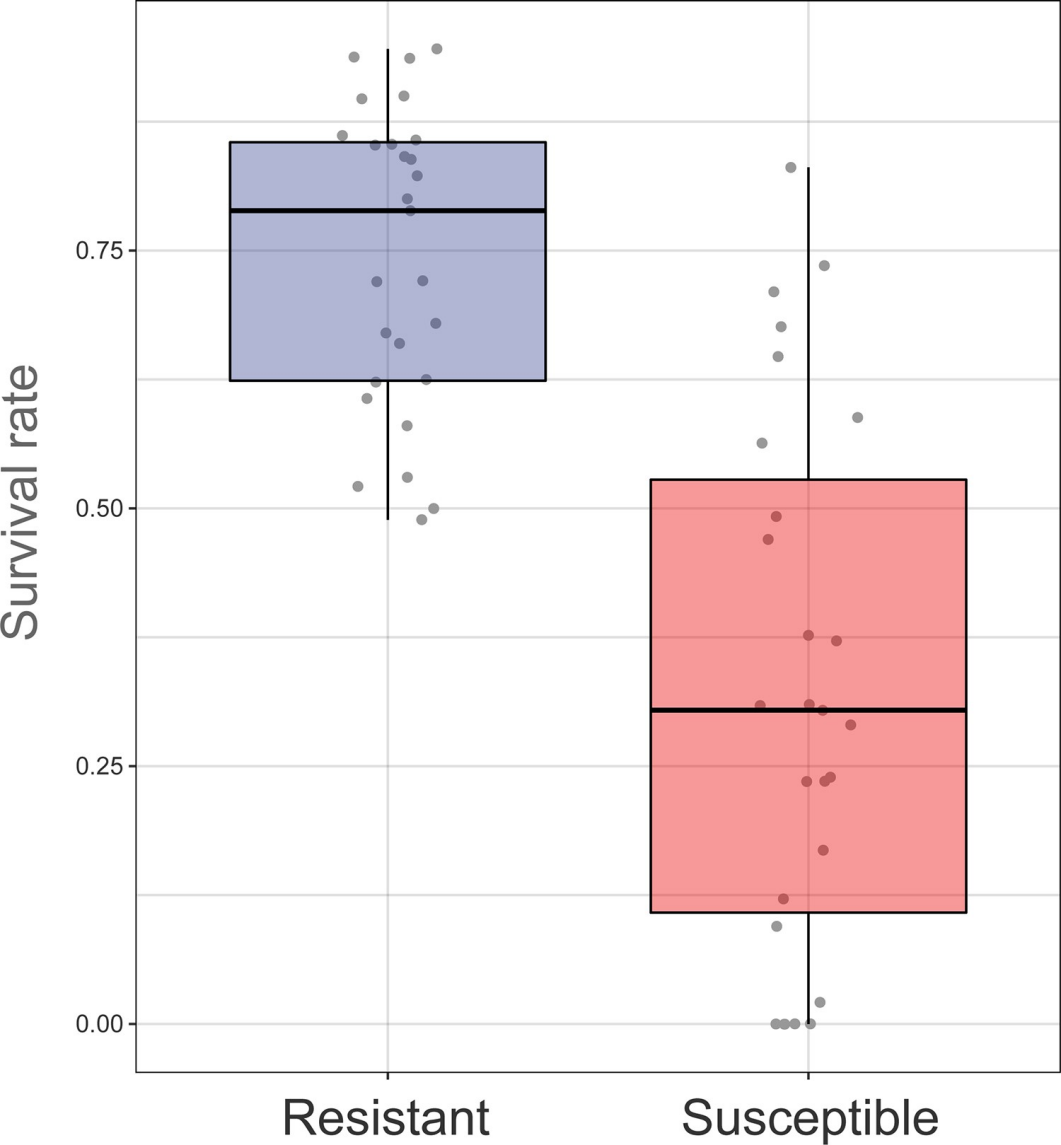
Survival rate of the resistant and susceptible families of *L*. *vannamei* after *VP*_*AHPND*_ infection. Significant differences were identified between the two types of families using Student’s t-test (*p* < 0.05, df = 52).

To learn the resistant mechanism of shrimp against vibriosis, we constructed resistant and susceptible families of *L*. *vannamei* under four generations of artificial selection. At least 200 full-sib families were used for the artificial selection each year. After four generations, 87 families (including resistant and susceptible families) were selected for the assessment of resistance to *VP*_*AHPND*_ (Fig A in S1 Text). Among the 87 families, ten resistant families with relatively high survival rates and ten susceptible families with relatively low survival rates were selected for subsequent studies. For convenience, the ten resistant families were collectively referred as the resistant family, and the ten susceptible families were collectively referred as the susceptible family hereafter. The two types of shrimp families (the resistant and susceptible families) exhibited differential performances in response to *VP*_*AHPND*_ infection. The resistant family had a significantly higher survival rate (74.28%) under infection than the susceptible family (32.55%, Student’s t-test, *p* < 0.05, df = 52)(Fig 1). In fact, the survival rate of the resistant family increased (from 47% in 2018 to 74% in 2021) after several generations of artificial selection [22,31]. This result indicated that the vibriosis-resistance trait was memorized and inheritable in the resistant family.

### A colonization resistance trait was shaped in the resistant family

**Fig 2:**
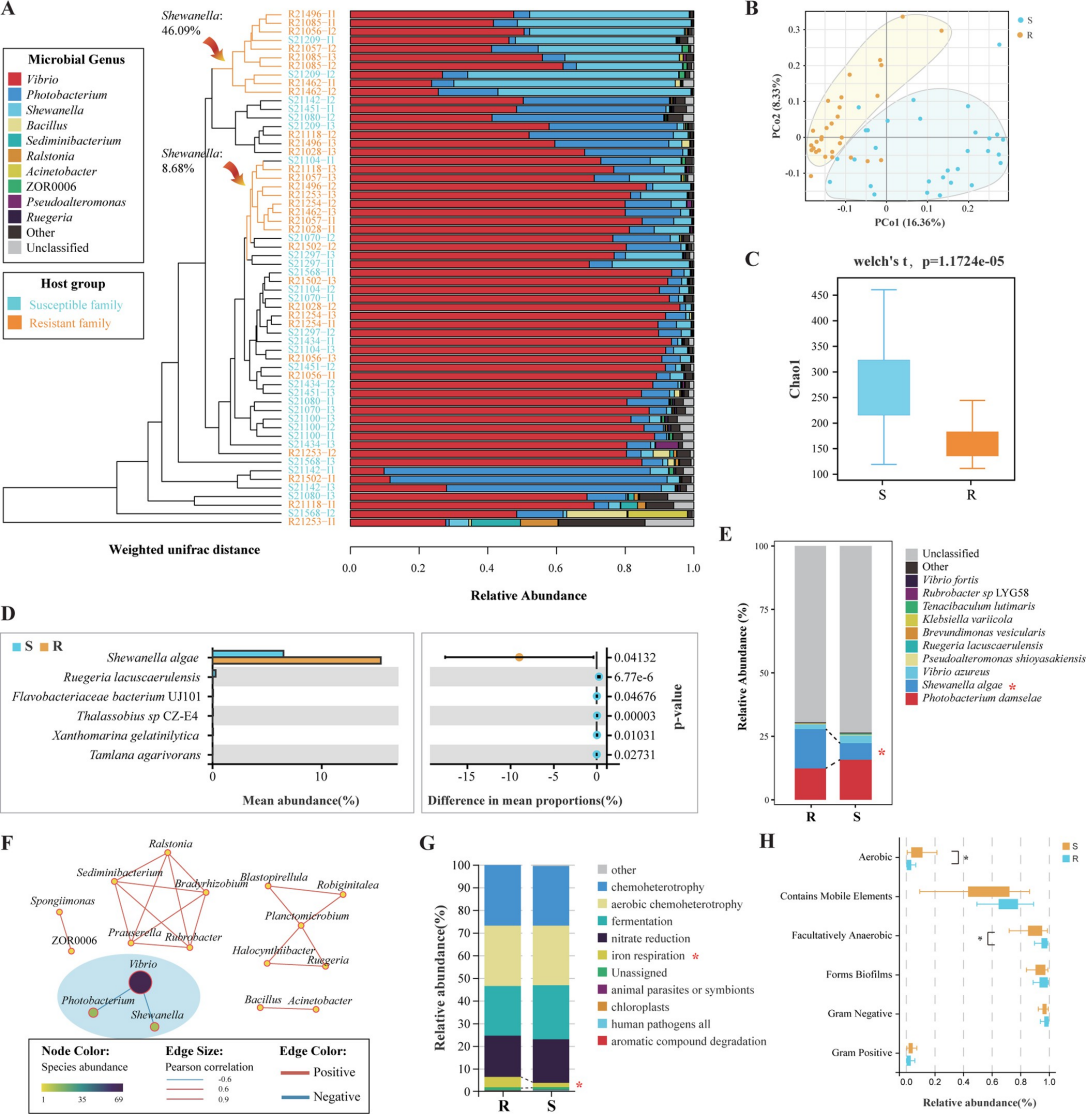
Intestinal microbial community composition and diversity in the resistant and susceptible families of *L*. *vannamei*. **(A)** Microbial community composition of the resistant and susceptible families. The unweighted paired-group method with arithmetic means (UPGMA)-based hierarchical clustering was used to cluster the samples according to the microbial community structure. **(B)** Principal component analysis of microbial community compositions in different samples. **(C)** Significant differences in microbial community structures between the two families (Welch’s t-test, *p* <0.05, df = 54). Welch’s t-test was used to detect the variation between the two families. **(D)** Welch’s t-test of the variation between the two families at the species level (Welch’s t-test, *p* <0.05, df = 50). **(E)** Microbial community structure in the two families at the species level. “*” indicates a significant difference in the content of *Shewanella algae* between the two families (Welch’s t-test, *p* <0.05, df = 50). **(F)** Microbial co-occurrence network of the two families. The network was built based on the relative microbial abundance in different samples using the Pearson coefficient as the distance measurement. The Fisher Z transformation was used to calculate a confidence interval (cor > 0.5) for Pearson’s correlation coefficient. The red line indicates a positive correlation between two genera, and the blue line indicates a negative correlation between two genera. **(G)** Relative abundance of each potential functional role in the intestinal microbiome between the two families. Microbial functional assemblages were predicted by FAPROTAX analysis. “*” indicates a significant difference (Welch’s t-test, *p* <0.05, df = 50) between the two families in terms of iron respiration. **(H)** Comparison of intestinal microbial phenotypes between the two families. The potential microbial phenotypes were predicted by BugBase. “*” indicates a significant difference (Welch’s t-test, *p* <0.05, df = 50) between the two families.

Since differential performances of the resistance of shrimp to *VP*_*AHPND*_ were detected between the two groups of families, we firstly performed microbiome sequencing to determine whether a colonization resistance had developed in the resistant family (Fig 2A). *Vibrio*, *Photobacterium*, and *Shewanella* were the three predominant genera identified in both families, accounting for more than 90% of the total intestinal microbes in most of the examined samples (57/60) (Fig 2A and Fig B in S1 Text). Significant differences in microbial community composition between the resistant and susceptible families were identified by PCA (Fig 2B) and alpha-diversity tests (Welch’s t-test, *p* < 0.01, df = 54; Fig 2C). Specifically, a significant deviation in abundance was observed between the two types of families for *Shewanella algae* and five other species (Welch’s t-test, *p* <0.05, df = 50; Fig 2D). Among these species, *S*. *algae* was the only one with a high abundance, and with a deviation of ~10% (9.34%) between the two types of families (Fig 2E and Table A in S1 Text). Thus, in contrast to the susceptible family, the resistant family has a superior capability in enriching *S*. *algae* in the intestine.

Notably, *S*. *algae* is a potential probiotic which can enhance the survival of *L*. *vannamei* after *VP*_*AHPND*_ infection, although its cytotoxicity and antimicrobial resistance have not yet been tested [32,33]. Dietary supplementation with *S*. *algae* strains can control the *Vibrio* load in *L*. *vannamei* [33]. Consistently, a strong negative correlation between *Vibrio* and *Shewanella* was found in the microbial co-occurrence network (Fig 2F). Therefore, the enrichment of a single beneficial species (*S*. *algae*) in the intestine of the resistant family contributed to the formation of colonization resistance, which should be a key and direct mechanism for the shrimp resistance to *Vibrio*.

The functional assemblages and potential phenotypes of the microbiomes in the two types of families were predicted by FAPROTAX and BugBase analyses, respectively (Fig 2G and 2H). Compared to those of the susceptible family, the microbiota in the resistant family had a relatively greater abundance of iron respiration, which were more facultatively anaerobic and less aerobic. *S*. *algae* is a typical dissimilatory iron-reducing bacterium, and iron plays an essential and special role in iron respiration in *Shewanella* spp. [34]. *S*. *algae* is a facultative anaerobic bacterium capable of utilizing oxygen and iron-oxides as the terminal electron acceptors for cell metabolism [35]. Therefore, the functional enrichment of iron respiration and facultatively anaerobic appear to be specifically needed by *S*. *algae* and contribute to its colonization.

### Differential gene expression profiles between the resistant and susceptible shrimp families

**Fig 3:**
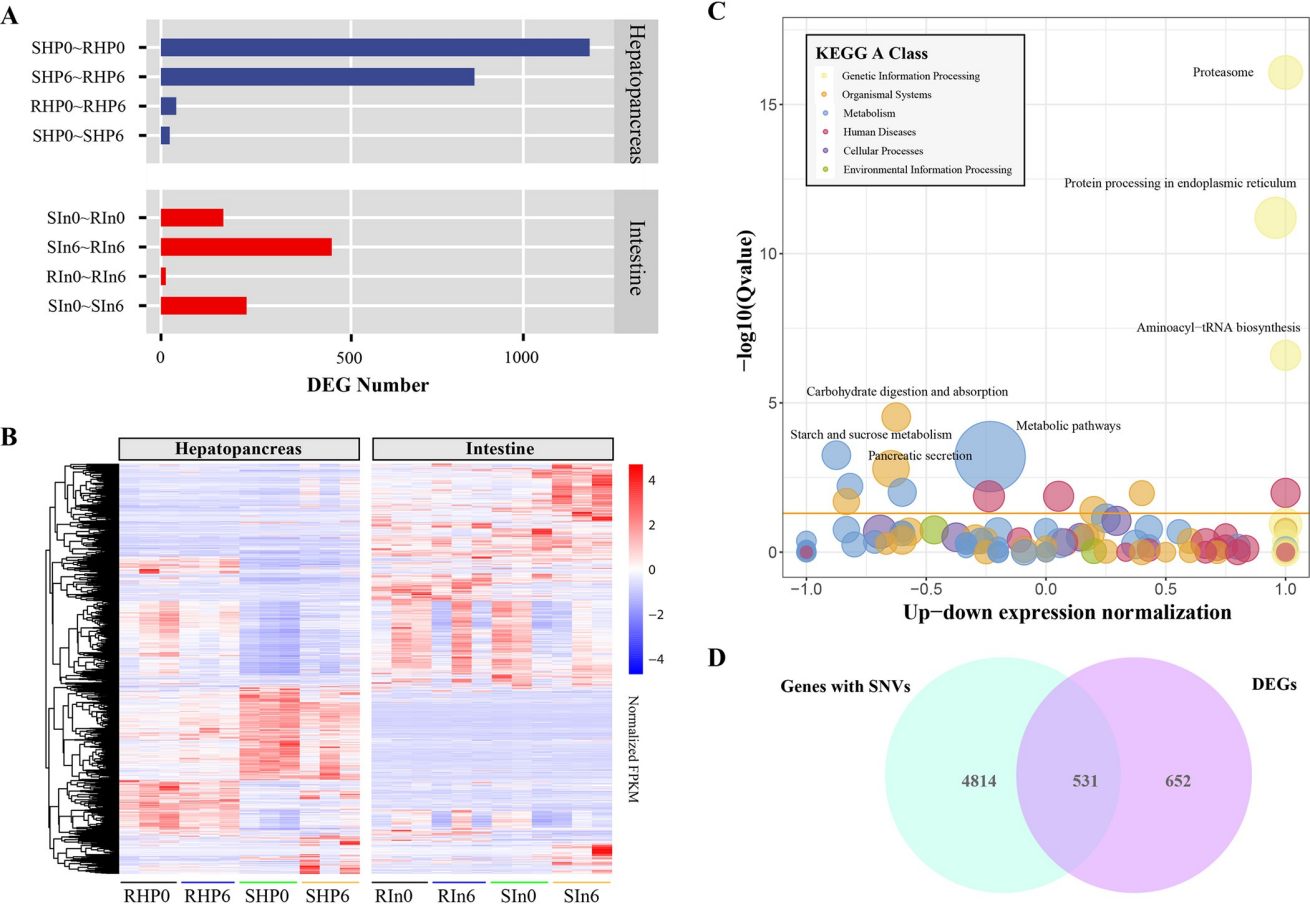
RNA-seq and differential gene expression analyses of the resistant and susceptible families. **(A)** Number of DEGs in pairwise comparisons. **(B)** Heatmap of the expression profiles of total DEGs. **(C)** KEGG enrichment of DEGs in the comparison between SHP0 and RHP0. **(D)** Venn diagram of genes with SNVs and DEGs in the comparison between SHP0 and RHP0.

From the aspect of the host, we first performed RNA-seq on hepatopancreas and intestinal tissues of the two types of families to determine their differential responses to infections at the transcriptional level. A large number of DEGs were identified in pairwise comparisons between the two families, as well as between pre- and post-infection (6 h post infection) (Fig 3A). Far more DEGs were identified primarily in the hepatopancreas than in the intestine, and far more DEGs were identified in the comparisons between the two families (SHP0-RHP0: 1183 DEGs and SHP6-RHP6: 805 DEGs) than in the comparisons between pre- and post-infection (RHP0-RHP6: 42 DEGs and SHP0-SHP6: 24 DEGs) (Fig 3A and 3B and Fig C in S1 Text). This result indicated that the two families had a stable difference in gene expression profiles regardless of the infection status of the shrimp (Fig D in S1 Text). Rather than the intestine, the hepatopancreas appears to be the primary site of the host immune response at the transcriptional level.

The 1183 DEGs from the comparative group of SHP0-RHP0 were primarily enriched in a number of pathways related to protein synthesis and processing, including the proteasome (hypergeometric test, adjusted *p* value = 8.53E-17), protein processing in endoplasmic reticulum (adjusted *p* value = 6.30E-12), and aminoacyl-tRNA biosynthesis (adjusted *p* value = 2.55E-07) (Fig 3C). Thus, the resistant family may differ from susceptible lines in the synthesis, processing, and metabolism of proteins.

Based on the RNA-seq data, we mapped the clean reads to the shrimp genome and identified 21,507 single nucleotide variations (SNVs that include SNPs and Indels), covering 5347 genes (S1 Data). More than 98% of SNVs (21,247) were heterozygous. The majority of SNPs were synonymous mutations (10,303), and a few were non-synonymous (2800). These genes with genetic mutations were majorly enriched in pathways of cell cycle, RNA transport, DNA replication, lysosome, and many other pathways (Fig E in S1 Text). Among these 5347 genes, 531 genes were DEGs that identified in the comparative group of SHP0-RHP0 (Fig 3D). Genetic mutations might be responsible for functional and/or transcriptional diversity in these genes.

### A group of genes were specifically activated in the resistant family through disruption of complete methylation

**Fig 4:**
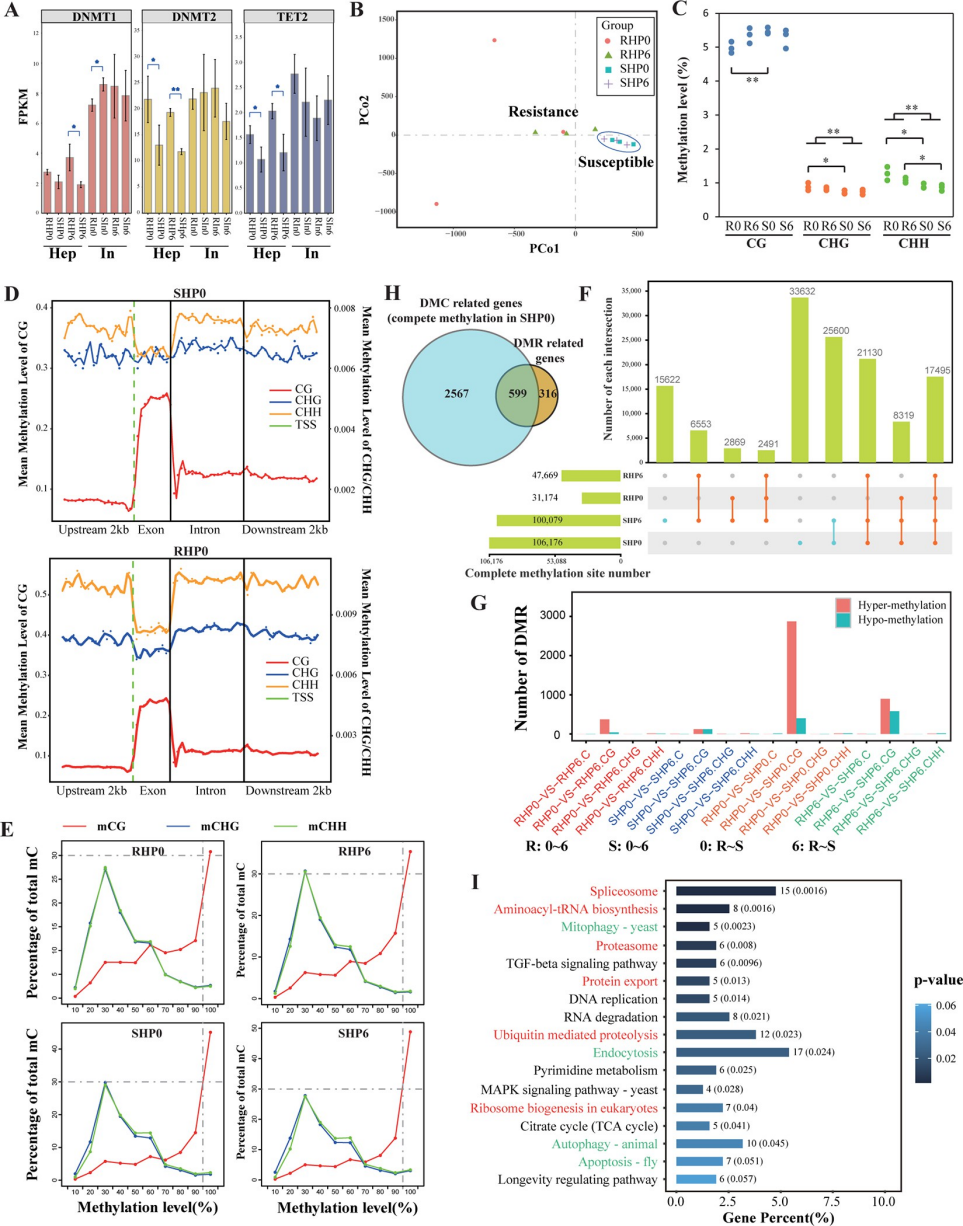
Differential DNA methylation between the resistant and susceptible families. **(A)** Differential expression of DNA methyltransferases (DNMT1 and DNMT2) and a DNA demethylase (TET2) in the hepatopancreas (Hep) and intestine (In) of the resistant (R) and susceptible (S) families. “*” indicates significant expression differences (Student’s t-test, *p* < 0.05, df = 4) between the two families in samples with (6 hours post infection) and without (0 h) *V*. *parahaemolyticus* infection. **(B)** Principal component analysis of the hepatopancreatic methylomes of the two families pre- and post-infection. **(C)** Methylation levels of 5-methylcytosine in different sequence contexts (CG, CHG, and CHH. H = A, C or T). Significance was tested between the two families (Student’s t-test, df = 4, * *p* < 0.05; ** *p* < 0.01). **(D)** Methylation profiles along the gene body in samples of the susceptible (SHP0) and resistant (RHP0) families pre-infection. **(E)** Distribution of the methylation levels of total mC. mC sites with methylation levels higher than 90% were considered as complete methylation sites (CMSs). **(F)** Venn diagram representing the intersection of CMSs in each sample. Blue dots indicate specific CMSs in the susceptible family. Red dots indicate the intersections of CMSs in the two families. **(G)** Counts of DMRs identified in each pairwise comparison. **(H)** Venn diagram representing DMR-related genes (DMGs in the comparison group of RHP0- SHP0) and DMC (sites that are completely methylated in SHP0 but partially methylated in RHP0) related genes. **(I)** KEGG enrichment of DMGs (RHP0-SHP0). DMGs were primarily enriched in protein synthesis and processing pathways (red) and endocytosis-related pathways (green).

Three DNA methylation-related genes encoding DNA methyltransferases (DNMT1 and DNMT2) and demethylases (TET2) were identified in the *L*. *vannamei* genome. The differential expression of these three genes between the two families was almost exclusively found in the hepatopancreas rather than the intestine (Fig 4A). This result suggested that DNA methylation is more active in the hepatopancreas than in the intestine, which was in line with the transcriptional activity in the hepatopancreas. Thus, hepatopancreas samples were selected for subsequent methylome sequencing and analysis.

Whole-genome bisulfite sequencing was subsequently conducted to profile the methylomes of the two families pre- and post-infection (Fig F and Table B in S1 Text). Whole-genome DNA methylation profiles were calculated and compared for both families (Fig 4B and 4C and Fig G in S1 Text). Among the three contexts of methylation (CpG, CHG, and CHH), CpG had the highest methylation level (5.72% on average) (Fig 4C). Methylated CpG sites were specifically concentrated in the exons of the gene body (25.32% on average) (Fig 4D and Figs H and I in S1 Text), whereas the methylation levels of CHH and CHG in the exons were only 0.71% and 0.74%, respectively. In addition, the Spearman correlation between gene expression and gene body methylation levels was higher (mean rho = 0.45) than that of other regions (mean rho values of 0.24 and 0.38 for upstream and downstream regions, respectively) (Figs J-L in S1 Text). Therefore, gene body exonic CpGs should be the major target of methylation regulation in gene expression in shrimp.

As indicated by PCA and hierarchical clustering analyses, the DNA methylation profiles were significantly biased between the two families (Student’s t-test, *p* < 0.05, df = 10). Whereas, no significant changes were found in the shrimp before and after infection (Fig 4B and Fig G in S1 Text). This revealed that the epigenetic variation between the two families was stably inherited and hardly affected by sudden environmental changes. Compared to the resistant family (4.98%), the susceptible family had significantly higher levels of CpG methylation (5.47%, Student’s t-test, *p* < 0.01, df = 4) (Fig 4C); thus, abundant differentially methylated sites (DMCs) were identified between them (60,331 DMCs in the comparison of RHP0-SHP0) (Fig M in S1 Text). Genomic sites were either completely methylated (CMSs, methylation level > = 90% herein) or partially methylated (PMSs, methylation level < 90% herein). Compared to the susceptible family (> 43.75% of mCpG sites were CMSs), the resistant family had fewer CMSs in both pre-infected (31.46%) and post-infected shrimp (35.68%) (Fig 4E). Generally, CMSs are more stable than PMSs in the genome [36]. Even in the case of *Vibrio* infection, most of the CMSs stayed in hyper-methylation levels (> 52%), with only 7.9% and 6.5% of the CMSs being differentially methylated (bias > 20%) in the resistant and susceptible families, respectively. Furthermore, it was astonishing to find that a large number of CMSs (74,854) were specifically present in the susceptible family, whereas no specific CMSs, not even one, were found in the resistant family (Fig 4F). This result indicated that a large number of CMSs have a tendency to be partially methylated in the resistant family. In the resistant family, many CpG sites broke the shackles of complete methylation (Fig 4E and Fig M in S1 Text), reflecting a more active epigenetic system.

Subsequently, we identified differentially methylated regions (DMRs) by pairwise comparisons to investigate regions with significant methylation changes (Pearson’s chi-square test, *q* ≤ 0.05). A total of 3273 and 1477 DMRs were identified between the two families pre- (RHP0-SHP0) and post-infections (RHP6-SHP6), respectively (Fig 4G). Among the 3273 DMRs identified in the comparison of RHP0-SHP0, the majority (2873 DMRs, 87.78%) were hyper-methylated in the susceptible family. Around these DMRs, 915 DMR-related genes (DMGs) were identified, and interestingly, most of them (599 genes, 65.46%) contained DMCs that were completely methylated in the susceptible family (SHP0) but partially methylated in the resistant family (RHP0) (Fig 4H). Taken these results together, compared with the susceptible family, the resistant family had relatively lower methylation levels, fewer CMSs, fewer hyper-methylated DMRs, and fewer hyper-methylated DMGs. Furthermore, these DMGs showed similar functional enrichment with that of the DEGs (Fig 4I). In such cases, abundant DMRs and associated DMGs were activated in the resistant family compared to the susceptible family, reflecting its superior activity in resistance of shrimp to *VP*_*AHPND*_.

### The major targets of DNA methylation regulation involved the pathways of protein synthesis and processing

**Fig 5:**
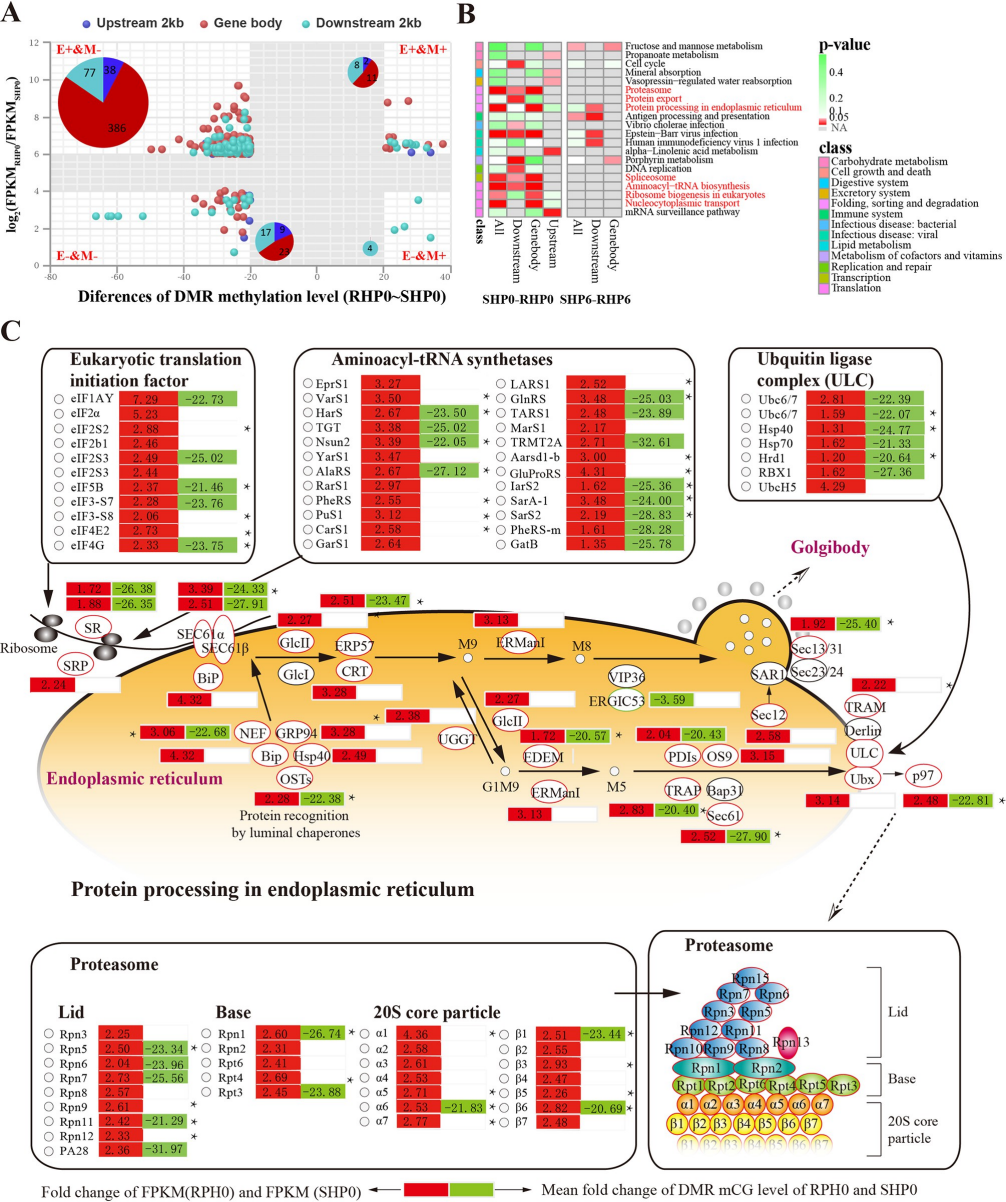
Correlation between DNA methylation and gene expression. **(A)** The correlation between DMGs and DEGs in the comparison group of RHP0-SHP0. Pie charts indicate the number of DEGs with DMRs in different regions of genes (gene body, 2 kb upstream and 2 kb downstream of the gene body). **(B)** KEGG enrichment of the intersections of DEGs and DMGs in each pairwise comparison. Only the KEGG terms with significant enrichment (hypergeometric test, *p* < 0.05) are shown. Comparisons in the groups of SHP0-SHP6 and RHP0-RHP6 are not shown due to the lack of significant enrichment. **(C)** DEGs and DMGs in the protein synthesis and processing pathways in the comparison group of SHP0-RHP0. * indicate the genes with SNVs.

In the comparison group of RHP0-SHP0, 245 genes (covering 575 DMRs) were involved in the intersection of DEGs and DMGs (Fig 5A). As expected, a strong negative correlation between DNA methylation and gene expression was found for these genes (Fig 5A). Notably, most of these genes (87.13%) were specifically hyper-expressed in RHP0, while their DMRs were hypo-methylated (Fig 5A and Fig N in S1 Text). Similar to the DEGs and DMGs, these 245 genes (intersection of DEGs and DMGs) were functionally enriched in pathways related to protein synthesis and processing, including aminoacyl-tRNA biosynthesis, ribosome biogenesis, nucleocytoplasmic transport, proteasome, protein export, protein processing in endoplasmic reticulum, and spliceosome (Fig 5B). In addition, abundant SNVs were also identified in many genes of these pathways (Fig 5C), suggesting that genetic and epigenetic mutations may both participate in regulating protein synthesis and processing procedures.

Aminoacyl-tRNA synthetases (AARS) covalently link an amino acid to a tRNA, which are crucial guarantors of the fidelity of protein synthesis [37]. Most genes (13 genes) encoding various AARS were hypo-methylated and hyper-expressed in the resistant family (Fig 5C). Similar results have been identified in genes encoding eukaryotic translation initiation factors (eIFs), which are key soluble proteins involved in facilitating translation initiation [38]. Newly synthesized proteins enter the endoplasmic reticulum (ER) undergoing a series of modifications and encounter a number of molecular chaperones and folding enzymes that work together to assist proteins in folding correctly before they are released from the ER [39]. The released proteins will be transferred to the proteasome, a highly sophisticated protease complex that cooperates with ubiquitin to selectively and processively hydrolyze client proteins [40]. Consistent with this, genes involved in protein processing in the ER and proteasome, including most molecular chaperones, ubiquitin ligase complex (ULC), and proteases of the proteasome, also appeared to be hypo-methylated and hyper-expressed in the resistant family (Fig 5C). Thus, the pathways of protein synthesis and processing might be the major targets of DNA methylation regulation, which appeared to be more active in the resistant family than in the susceptible family.

### Host DNA methylation regulates the colonization resistance of the intestinal microbiota

**Fig 6:**
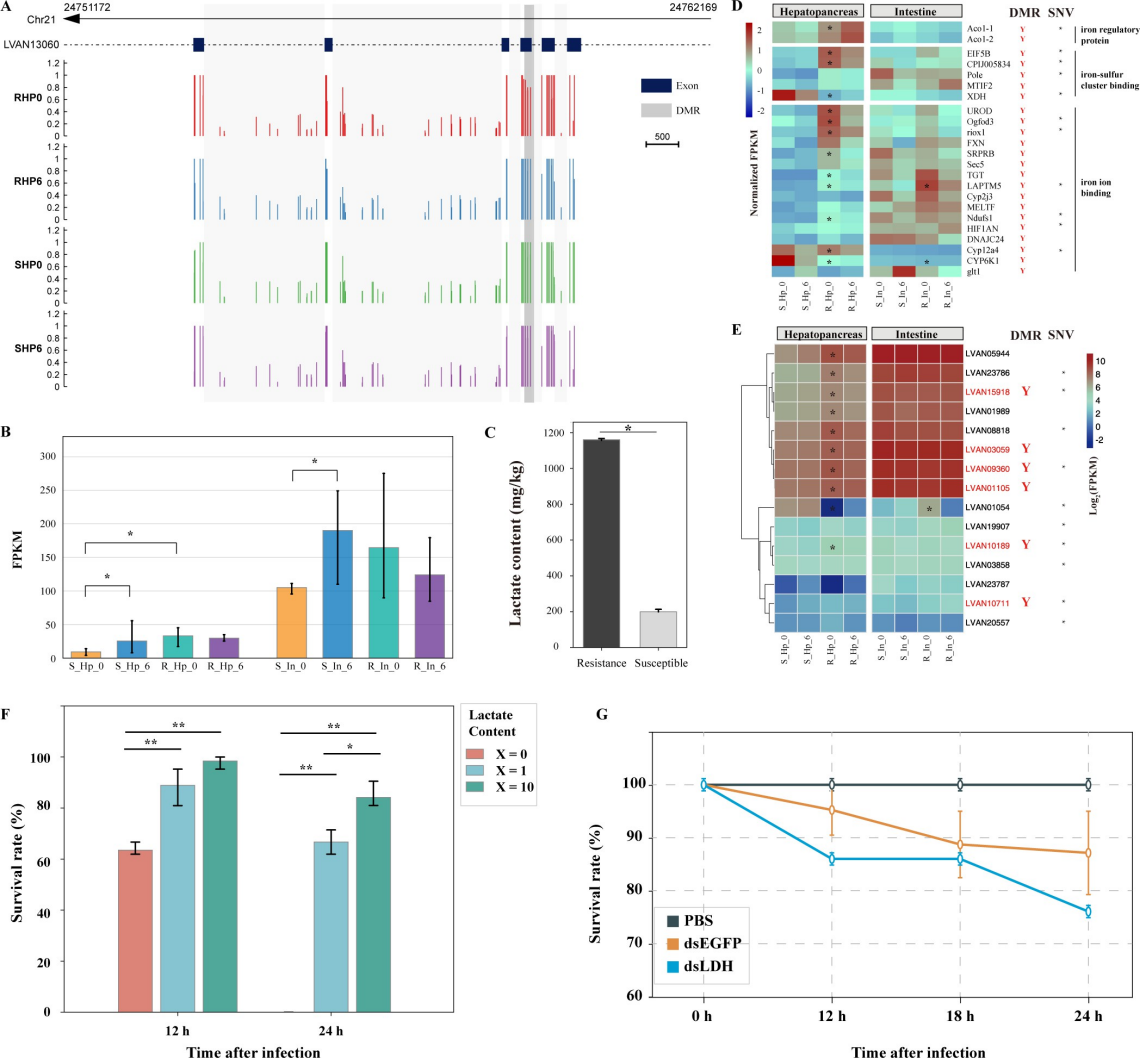
Association between host DNA methylation and the intestinal microbiota. **(A)** DNA methylation profile of the gene encoding lactate dehydrogenase (LDH). A DMR (RHP0-SHP0, yellow background) was found in the third exon of the gene. **(B)** Expression profiles of *LDH* in the hepatopancreas and intestine of the two families. * indicates a significant difference (Student’s t-test, *p* < 0.05, df = 4) at expression levels between the two families. **(C)** Significant difference (Student’s t-test, *p* < 0.05, df = 4) of lactate content in the intestines of the two families. The intestinal lactate content was determined by HPLC. **(D)** Expression profiles of iron regulatory and binding genes in the hepatopancreas and intestine of the two families. * indicates a significant difference in expression (Student’s t-test, *p* < 0.05, df = 4) between RHP0 and SHP0. “Y” indicates that the genes contain DMRs; * indicates the genes with SNVs. **(E)** Expression profiles of cytochrome c genes in the hepatopancreas and intestine of the two families. **(F)** Survival rates of *VP*_*AHPND*_-challenged shrimp fed diets supplemented with different concentrations of lactate. The weights of the basal diet, egg white and lactate follows ratios of 119: 5: X, where X = 0, 1, and 10. * (Student’s t-test, *p* < 0.05, df = 4) and ** (*p* < 0.01) indicate significant differences between two comparison groups. **(G)** Survival rates of shrimp that injected with PBS, dsEGFP, or dsLDH under *VP*_*AHPND*_ challenge.

As indicated above, the enrichment of the probiotic *S*. *algae* plays a pivotal role in forming colonization resistance. The carbon source of many *Shewanella* strains is quite restricted, mainly fermentation end products such as lactate, certain amino acids, and hydrogen [41]. Intriguingly, lactate, but not other conventional carbon sources used to support growth, is able to promote iron uptake in *Shewanella* spp. [34]. Thus, the abundance of lactate and iron in the host intestine may provide a proper niche for the colonization of *S*. *algae*. Lactate formation is specifically catalyzed by lactate dehydrogenase (LDH), an important enzyme in the anaerobic metabolic pathway [42]. Only one gene (*LDH*) encoding LDH was identified in the *L*. *vannamei* genome. No SNVs were identified in the gene body of *LDH*. In contrast, a DMR was found in the region of the third exon, which was partially methylated in RHP0 (the pre-infected resistant family) but completely methylated in RHP6 (the post-infected resistant family) and the susceptible family (Fig 6A). The expression level of *LDH* was significantly higher in RHP0 than in SHP0 (Student’s t-test, *p* < 0.05, df = 4, Fig 6B). Similar results have also been found in the intestine. It indicated that the transcriptional diversity of *LDH* might be a result of epigenetic remodeling rather than genetic mutation. As detected by HPLC, the intestinal lactate content of the resistant family (1160.41 mg/kg) was significantly higher than that of the susceptible shrimp (199.98 mg/kg, Student’s t-test, *p* < 0.05, df = 4) (Fig 6C). In addition, shrimp fed with lactate have significantly higher survival rate than those fed with basal diet under *VP*_*AHPND*_ challenges (Student’s t-test, *p* < 0.01, df = 4), and consistently, *LDH* knockdown resulted in a decrease in shrimp survival rate (Fig 6F and 6G). Therefore, the enrichment of lactate in the intestine of the resistant family should be the direct causes for the *S*. *algae* accumulation and colonization resistance formation, which appears to be regulated by the host epigenetic system.

Iron plays an essential and specific role in the iron respiration of *Shewanella* spp., and this respiration process relies on a large number of c-type cytochromes [34]. A large number of genes related to iron homeostasis and transport were identified in the host genome, including those encoding iron regulatory proteins, iron-sulfur cluster binding proteins, iron ion binding proteins, and cytochrome c (Fig 6D and 6E). Interestingly, many of these genes were both hypo-methylated and hyper-expressed in the hepatopancreas of the resistant family, which contained SNVs as well (Fig 6D). Among the 15 cytochrome c genes, 10 were differentially expressed in RHP0 in comparison to SHP0, and five of these 10 genes were DMGs (Fig 6E). Therefore, the host might also regulate endogenous iron homeostasis through the epigenetic system, which is necessary for iron respiration in *S*. *algae*.

### Methylation inhibition causes enrichment of *S*. *algae* in the intestine

**Fig 7:**
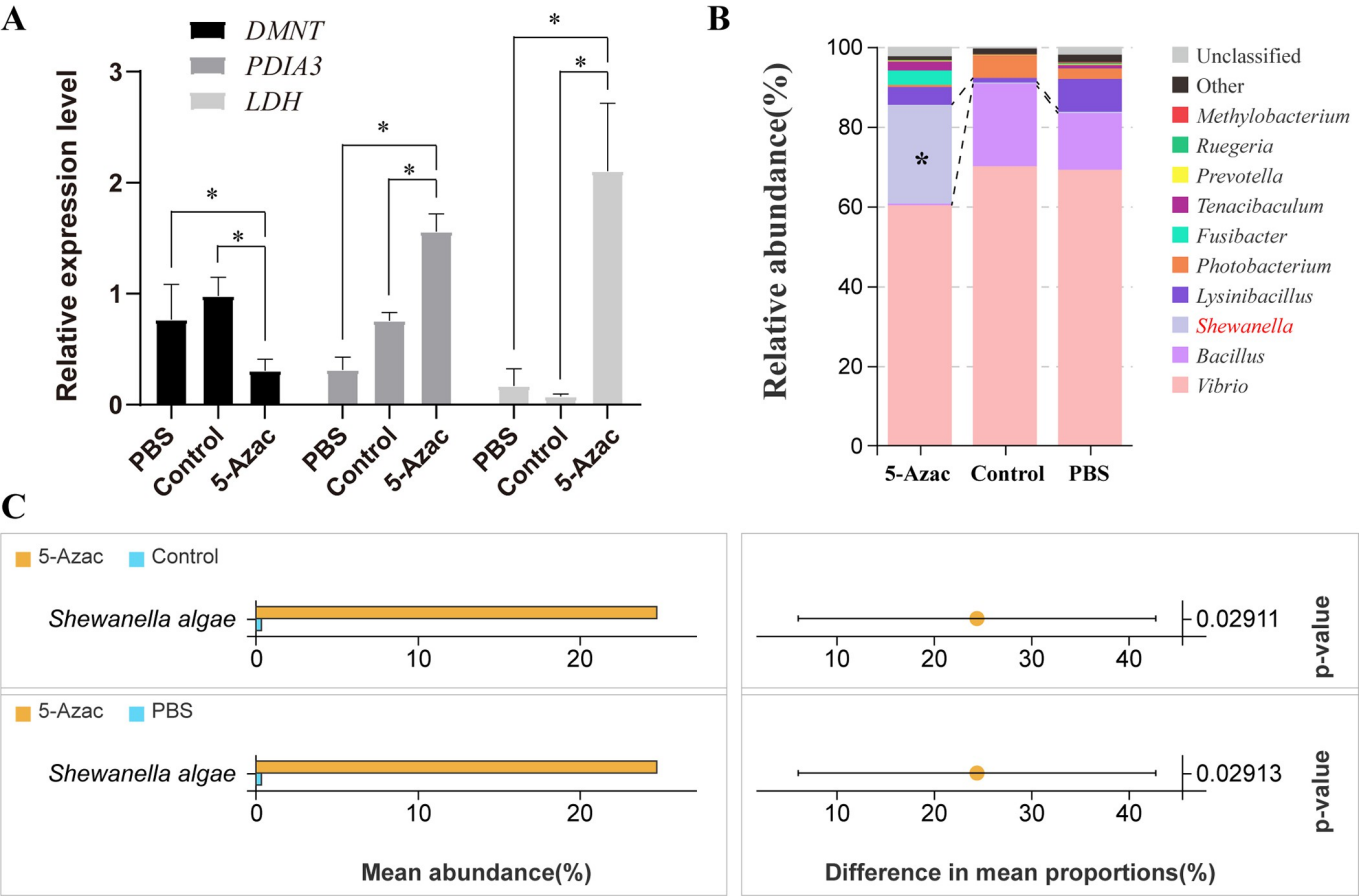
Effects of 5-Azac treatment on the intestinal microbial community. (**A**) The relative expression levels of three genes (*DMNT*, *PDIA3*, *LDH*) after treatment with 5-Azac and PBS for five days. Samples before treatment (0 d) were set as the control group. * indicates a significant difference (*p* <0.05, df = 5) according to Student’s *t* test. (**B**) Microbial community structure in the three groups at the genus level. * indicates a significant difference in the content of *S*. *algae* between the 5-Azac group and the other two groups (Welch’s t-test, *p* <0.05, df = 5). (**C**) Welch’s t-test of the variation between each pair of groups at the species level.

To test the functions of methylation in shaping colonization resistance, we injected the DNA methylation inhibitor 5-Azac into normal shrimp. We hypothesized that the intestinal microbial community would be reconstructed if DNA methylation was inhibited. After treatment with 5-Azac, the expression of the methyltransferase gene *DMNT* was down-regulated, while the expression of the gene encoding LDH was significantly up-regulated (Student’s t-test, *p* < 0.05, df = 4 Fig 7A). Similarly, genes involved in protein processing (disulfide-isomerase, *PDIA3*) were also upregulated in the 5-Azac treatment group. This result suggested that the inhibition of methylation can activate the activity of LDH, and protein synthesis and processing related genes, in line with the findings in the resistant family. Since LDH was up-regulated, we wondered whether it can induce enrichment of *S*. *algae* in the intestine. Thus, we examined the intestinal microbial community in the 5-Azac treatment group. As expected, we found that *S*. *algae* was significantly more enriched in the 5-Azac group than that in the control groups (Welch’s T test, *p* < 0.05, df = 5; Fig 7B and 7C). Notably, *S*. *algae* was the only species with a significant bias in the abundance of the compared groups (Fig 7C). In contrast, the abundance of *Vibrio* was relatively reduced in the 5-Azac group (5-Azac: 60.25%, Control: 70.04%, and PBS: 69.13%, Fig O in S1 Text). This result indicates that methylation inhibition can result in the enrichment of *S*. *algae* and a reduction in *Vibrio* abundance.

## Discussion

### Enrichment of a single probiotic species shapes a defense mechanism of colonization resistance for shrimp against *Vibrio* infections

The intestinal microbiota is essential for host growth and survival [43,44]. They aid in digestion and energy homoeostasis, prevent colonization of infectious agents, and help maintain host mucosal immunity [44,45]. In aquaculture, the enrichment of probiotics in the intestine appears to be a crucial mechanism for host resistance to pathogens. For instance, the probiotics, Phaeobacter and Cyanobacteria, are enriched in the pathogen-resistant families of the Chinese tongues sole and oysters [26,46]. In this study, we found that a single probiotic species, *S*. *algae*, was significantly enriched in the intestine of the resistant family of *L*. *vannamei*.

*S*. *algae* has increasingly been used as a potential probiotic in aquaculture since it has been identified to inhibit or reduce the pathogenicity of *Vibrio* in penaeid shrimp farming [33,47]. Feeding with incorporated *S*. *algae* strain could effectively control the *Vibrio* load and modulate host immune genes in *L*. *vannamei*, resulting in an improved shrimp survival rate and growth [32,33]. A negative relationship was also identified between *Vibrio* and *S*. *algae* in this study. Therefore, rather than the host immune response, enrichment of *S*. *algae* in the intestine should be a direct way for vibriosis resistance in *L*. *vannamei*. Although *S*. *algae* is beneficial to shrimp, it needs to be tested for pathogenicity, antimicrobial resistance, and potential ecological impacts before it is released into the environment, because it includes pathogenic strains that may cause harm to humans and some animals.

In fish, the intestinal microbiome has been proposed to control host immunological homeostasis and inflammatory responses through a microbe-intestine-immunity axis, thereby enhancing resistance to vibriosis [26]. In this study, we found that a defense mechanism of colonization resistance was adopted by shrimp resistant to vibriosis, where *S*. *algae* accumulated to prevent the expansion of *Vibrio*. With the development of colonization resistance, the intestinal environment has changed from aerobic to facultative anaerobic, with increased levels of lactates and iron, which is unsuitable for *Vibrio* growth but instead favors the growth of *S*. *algae*. In addition, the probiotic potential of *S*. *algae* in modulating immune-related genes is considered as a tool to control *VP*_*AHPND*_ infection in shrimp [32].

### The epigenetic system memorizes and regulates colonization resistance

AHPND/EMS, caused by *VP*_*AHPND*_, has led to a devastating impact on global shrimp farming [48]. Thus, great efforts have been made to understand the molecular basis of disease resistance [19]. Both genetic and epigenetic factors may be drivers of resistance trait formation. Genetic basis studies have focused on the identification of QTLs and causative genes controlling disease resistance or susceptibility [15,18–22]. Hundreds of SNPs related to resistance have been identified, and a number of targeted proteins have been proposed for *L*. *vannamei* [18,21]. As expected, many innate immune genes, including those encoding anti-lipopolysaccharide factor, ubiquitin carboxyl-terminal hydrolase, PI3K regulatory subunit, Toll-like receptors, and hemocyanin, have been proposed to be targets for resistance in association studies [18,21]. Whereas, it remains unclear whether these genes play a key role in resistance, and no relationship has been found between them and colonization resistance. In this study, abundant SNVs were also identified in the two shrimp families, and genes with SNVs had a broad functional distribution (Fig E in S1 Text). However, it is difficult to test whether these genetic mutations are responsible for the development of resistance trait at present. Further genome resequencing and genome-wide association studies (GWAS) will help to elucidate the functional roles of these genetic mutations.

Environmental regulation of aquaculture traits appears to be mediated at the level of epigenetic regulation [19]. In this study, robust evidence indicated that epigenetic factors may play a key role in shrimp resistance to vibriosis through methylation regulation on colonization resistance. The significant bias in methylation patterns between the two families was stable and barely affected by pathogen infection. A large number of sites and targeted genes were specifically activated in the resistant family. These findings indicated that artificial selection and breeding have induced epigenetic remodeling in shrimp, which were memorized and transgenerationally inherited. Hypo-methylation and hyper-expression of genes related to LDH and iron homeostasis provide rich sources of specific carbon (lactate) and ions for iron respiration in *S*. *algae*. This directly led to the enrichment of *S*. *algae* in the resistant family, which thus effectively reduced the intestinal *Vibrio* load. It suggested that colonization resistance was memorized as epigenetic information in shrimp, which promoted resistance against *VP*_*AHPND*_. This reprogrammed epigenetic information might be selected in the resistant family and cause the formation of disease resistance trait. In turn, it may also be a result of intestinal microbiota mediation. The intestinal microbes can produce a myriad of metabolites; the changes in microbes and related metabolites will mediate host epigenetic programming [49,50].

In addition, pathways related to protein synthesis and processing were also the major targets for methylation regulation. However, the functional role of protein synthesis and processing in the resistance of shrimp to *Vibrio* has never been reported before. Nevertheless, these pathways are closely related to resistance to various stresses [15,51–59]. DEGs from the proteasome, protein processing in endoplasmic reticulum, ribosome, and aminoacyl-tRNA biosynthesis pathways were significantly enriched in response to stresses such as pathogen infection [55,58], heat shock [51], and dietary succinate [52]. In addition, in response to salinity stress [54,59], alkalinity stress [57], hazard stress [56], and nitrite stress [53], pathways of aminoacyl-tRNA biosynthesis, biosynthesis of amino acids, and ribosome biogenesis, are also actively regulated. Therefore, it can be concluded that the regulation of pathways related to protein synthesis and processing plays a crucial role in stress resistance. The hepatopancreas, as an integrated organ of immunity and metabolism, is involved in the detection and clearance of pathogens, antigen processing during acute virus infections, and infection-induced metabolic changes [60]. Upregulation of protein synthesis and processing may contribute to the enhancement of hepatopancreatic antimicrobial and metabolic functions.

### The hepatopancreas-intestine axis forms a barrier to *Vibrio* in shrimp

The hepatopancreas and intestine are two digestive organs with connections in shrimp. Besides, they also play important roles in the immunity of shrimp, and are critical to the overall health of the organism [61]. From our results, the hepatopancreas displayed significant differential expression patterns in comparison with that of the intestine, which suggested that the two tissues played different roles in resistance to pathogens. The intestinal barrier of shrimp is associated with immune proteins and a stable microbiota [62]. The intestinal microbiota is an important component of the hepatopancreas-intestine axis [63]. The hepatopancreas is an integrated organ of immunity and metabolism, that is capable of degrading and metabolizing toxic substances, as well as preventing bacterial metabolites from entering the circulation [60,63]. Impaired hepatopancreatic function leads to significant changes in the intestinal microbial community, which in turn affects intestinal barrier function [64]. In this study, we found significant changes in the epigenetic system of the hepatopancreas and apparent differences in the intestinal microbiota between the two families. The cooperation of the hepatopancreas and intestine formed an efficient barrier against *Vibrio* in shrimp.

Intestinal colonization is an important ability for probiotics to exert beneficial effects *in vivo* [65]. The colonization of *S*. *algae* in the host intestine is a prerequisite for its probiotic function in shrimp. Lactate and iron are two components essential for the survival and growth of *S*. *algae*, because they are specialized carbon sources and ions needed for iron respiration [34,41]. As indicated by this study, the LDH gene, which is specialized for lactate production, was hypo-methylated and hyper-expressed in the resistant family. Similar results were observed for genes related to iron homeostasis and transport, including cytochrome c, iron regulatory proteins, iron binding proteins, and ferritin. Ferritin is an iron-scavenging protein with a key function in maintaining iron homeostasis, and is also an immune molecule produced by the hepatopancreas [60]. Lactate, but not other carbon sources, is able to promote iron uptake [34]. The intestinal lactate content was significantly higher in the resistant family than in the susceptible family. In addition, the survival of lactate-fed shrimp challenged with *VP*_*AHPND*_ was significantly higher than that of shrimp fed a basal diet, whereas *LDH* knockdown led to a decrease in shrimp survival. On this basis, the host provides a suitable environment for the colonization of *S*. *algae* and thus shapes a colonization resistance trait. In addition, methylation inhibition with the treatment of 5-Azac can result in the upregulation of LDH and the enrichment of *S*. *algae* in the host intestine. Therefore, changes in the hepatopancreas epigenetic system may contribute to the colonization of *S*. *algae* in the intestine, thus shaping colonization resistance.

In turn, the intestinal microbiota may participate in modulating the immune system of the hepatopancreas. Among the numerous health benefits of probiotics, modulation of the immune system is one of the most common purported benefits of probiotics, and their potency to stimulate systemic and local immunity under *in vitro* and *in vivo* conditions is noteworthy [45]. The epithelial cells of the hepatopancreas are a major source of immune molecules, and many of the corresponding immune genes were differentially expressed in the resistant family. Thus, enrichment of the probiotic *S*. *algae* may contribute to the generation of immune molecules in the hepatopancreas. Taken together, there is a crosstalk between the hepatopancreas and the intestine, which forms a barrier axis for resistance to *Vibrio*.

## Materials and methods

### Ethics approval and consent to participate

The methods were performed in accordance with relevant guidelines and regulations and approved by the Animal Ethics Committee [2020(37)] at the Institute of Oceanology, Chinese Academy of Sciences.

### Resistant and susceptible family sample collection

The selective breeding of the resistant and susceptible families of *L*. *vannamei* and assessment of their resistance were performed according to previous studies [15,22]. In brief, at least 200 full-sib families of *L*. *vannamei* derived from different breeding lines were constructed under the same conditions and selected for the assessment of resistance to *Vibrio* each year since 2017. The full-sib families were produced and stocked separately in Hainan Grand Suntop Ocean Breeding Co., Ltd., Wenchang, China. In order to identify the resistance to AHPND, the shrimp families were evaluated for their resistance by *VP*_*AHPND*_ challenge each year, and the families with high survival rates were mated to produce the next-generation families. The *VP*_*AHPND*_ strains were isolated from diseased shrimp in our laboratory and identified as *V*. *parahaemolyticus* as described in our previous study [66]. For the *VP*_*AHPND*_ challenge experiments, shrimp from each family were subjected to *VP*_*AHPND*_ immersion infection at a *VP*_*AHPND*_ concentration of 5×10^6^ CFU/ml. Then, the mortality of each family was checked every 2 h for 72 h. After selection for four generations, a total of 87 families, including the resistant and susceptible families, were selected for evaluation of resistance (Fig A in S1 Text). Among them, ten resistant families with high survival rates and ten susceptible families with relatively low survival rates were selected for this study, representing the resistant and susceptible families, respectively. To ensure the level of resistance in the two families, 54 groups of individuals from the ten resistant families and ten susceptible families were randomly selected and challenged with *VP*_*AHPND*_ again, and the results were similar to those of the previous challenge experiment. The mean body weights of the resistant and susceptible families were 1.95 ± 0.23 g and 2.01 ± 0.32 g, and their average survival rates were 74.28% and 32.55%, respectively.

For microbiome sequencing, intestinal samples were collected from 180 individuals from each of the resistant and susceptible families (each with ten full-sib families). Six individuals within a single full-sib family were pooled as one sample, and thus each full-sib family contained three replications. For transcriptome sequencing, individuals from both resistant and susceptible families received a second immersion of *VP*_*AHPND*_ for six hours. Hepatopancreas and intestines were collected from nine individuals from resistant (R4345) and susceptible (S4383) families both pre- and post-infections, and three individuals were pooled as one sample, so that each family contained three repeated samples. For DNA methylome sequencing, the hepatopancreases of the same samples used for RNA-seq were collected. All the samples were rapidly frozen in liquid nitrogen and stored at -80°C for subsequent sequencing programs.

### Microbiome sequencing and analysis

The intestine samples from resistant and susceptible families were used for microbiome sequencing. Microbial DNA was extracted using HiPure Stool DNA Kits (D3141-02, Magen, Guangzhou, China) according to the manufacturer’s protocols. The 16S rDNA target region of the ribosomal RNA gene was amplified by PCR. Amplicons were extracted from 2% agarose gels and purified using the AxyPrep DNA Gel Extraction Kit (AP-GX-500, Axygen Biosciences, Union City, CA, U.S.) according to the manufacturer’s instructions and then quantified using the ABI StepOnePlus Real-Time PCR System (Life Technologies, Foster City, USA). Purified amplicons were pooled in equimolar amounts and sequenced on the Illumina HiSeq platform.

Raw sequencing reads were filtered using FSATP (v0.18.0) [67]. Paired-end clean reads were merged into raw tags using FLASH (v1.2.11) with a minimum overlap of 10 bp and a maximum mismatch error rate of 2% [68]. Then, noisy sequences of the raw tags were filtered under specific filtering conditions to obtain high-quality clean tags. Clean tags were then clustered into operational taxonomic units (OTUs) with ≥ 97% similarity using the UPARSE (v9.2.64) pipeline [69]. All chimeric tags were removed using the UCHIME algorithm [70]. The tag sequence with the highest abundance was selected as a representative sequence within each cluster.

The representative OTU sequences were classified into organisms by a naive Bayesian model using the RDP classifier (v2.2) based on the SILVA database (v138.1), with a confidence threshold of 0.8 [71,72]. Abundance statistics for each taxon were visualized using Krona (v2.6) [73]. Stacked histograms of community composition were visualized using the R project ggplot2 package (v2.2.1) [74]. Pearson correlation analyses of species were calculated by the R project psych package (v1.8.4). Correlation coefficient networks were generated using the Omicsmart Tool (http://www.omicsmart.com).

Species comparisons between groups were performed using Welch’s t-test and the Wilcoxon rank test in the R project Vegan package (v2.5.3). For beta diversity analysis, sequence alignment was performed using Muscle (v3.8.31), and a phylogenetic tree was constructed using FastTree (v2.1) [75,76]. Then, weighted and unweighted Unifrac distance matrices were generated by the GuniFrac package from the R project.

Microbiome phenotypes of bacteria were categorized using BugBase [77]. The FAPROTAX database (Functional Annotation of Prokaryotic Taxa) was used to generate ecological functional profiles of bacteria [78]. The Pearson correlation coefficient between environmental factors and species was calculated using the R project psych package. Heatmaps and networks of correlation coefficients were generated using the Omicsmart tool.

### Transcriptome sequencing and analyses

Hepatopancreas and intestine samples collected pre- (0 h) and post-infection (6 h) from the resistant (RHP0, RHP6, RIn0, and RIn6) and susceptible (SHP0, SHP6, SIn0, and SIn6) families were used for transcriptome sequencing. Total RNA from these samples was extracted using TRIzol reagent kit (15596018, Invitrogen, Carlsbad, CA, USA) according to the manufacturer’s instructions. RNA purity and concentration were determined by an Agilent 2100 Bioanalyzer (Agilent Technologies, USA). Transcriptome libraries were then prepared using the TruSeq RNA Library Prep Kit (FC-122-1001, Illumina, San Diego, USA). The libraries were sequenced on the Illumina HiSeq2500 platform.

Based on the *L*. *vannamei* reference genome [79], the sequencing reads were mapped, and gene expression levels were calculated as fragments per kilobase of million fragments mapped reads (FPKM) values. Differential gene expression (DGE) analyses were conducted in the comparisons of the resistant and susceptible families (comparison groups included RHP0-SHP0, RHP6-SHP6, RIn0-SIn0, and RIn6-SIn6) and in the comparisons of pre- and post-infections (comparison groups included RHP0-RHP6, SHP0-SHP6, RIn0-SIn6, and SIn0-SIn6) by using DESeq2 [80]. Genes with a false discovery rate (FDR) < 0.05 and an absolute value of log2 of fold change (FC) > 1 were considered differentially expressed genes (DEGs). GO function and KEGG pathway enrichment analyses of DEGs were conducted by OmicShare Tools (www.omicshare.com/tools).

Based on the reads mapping results, the GATK (version 4.3.0.0) was used for calling variants of transcripts [81], and ANNOVAR was used for SNVs (including SNPs and InDels) annotation using default parameters [82]. The SNVs were further filtered when the resistant and susceptible families had different genotypes, and at least five samples (a total of six samples) shared the same genotype in each family. The function, genome site and type of variation of SNPs were also analyzed.

### DNA methylome sequencing and analysis

After extraction of genomic DNA from the samples, DNA concentration and integrity were examined by spectrophotometer (IMPLEN, CA, USA) and agarose gel electrophoresis, respectively. Then, DNA libraries for bisulfite sequencing were prepared. Briefly, genomic DNA was fragmented into 100–300 bp fragments by Sonication (Covaris, Massachusetts, USA) and then purified by MiniElute PCR Purification Kit (28006, QIAGEN, MD, USA). End repair was performed on the fragmented DNAs, and a single “A” nucleotide was added to the 3’ end of the blunt fragment. Then, the genomic fragments were ligated to methylated sequencing adapters. The fragments with adapters were bisulfite converted using the Methylation-Gold kit (FC-122-1001, ZYMO, CA, USA), and unmethylated cytosine was converted to uracil during sodium bisulfite treatment. Finally, the converted DNA fragments were amplified by PCR and sequenced using the Illumina HiSeq platform of Gene Denovo Biotechnology Co. (Guangzhou, China).

Raw sequencing reads were filtered by removing reads with more than 10% N and removing reads with low quality (40% bases with Q-value ≤ 20). Clean reads were then mapped to the *L*. *vannamei* genome using BSMAP (v2.90) with default parameters [83]. For each sequence context (CpG, CHG and CHH), methylation levels were calculated based on the percentage of methylated cytosine for the whole genome, for each chromosome, and for different regions of the genome (including the gene body, exons, introns, 2 kb upstream, 2 kb downstream, and transposable elements). To assess the different methylation patterns in different genomic regions, the methylation profiles of the flanking 2 kb regions and gene bodies were plotted based on the average methylation level in each window. Principal component analysis (PCA) was performed using the R package gmodels (http://www.r-project.org/).

Differential DNA methylation at each locus between the two families (RHP0-SHP0 and RHP6-SHP6) and between pre- and post-infection (RHP0-RHP6 and SHP0-SHP6) was determined using Pearson’s chi-square test in methylKit (v1.7.10). To identify differentially methylated cytosines (DMCs), the minimum read coverage to call a methylation status for a base was set to 4. Differentially methylated cytosines for each sequence context (CpG, CHG and CHH) were identified between each of the two samples. To identify differentially methylated regions (DMRs) between two samples, the minimum read coverage to call a methylation status for a base was set to 4. The DMRs for each sequence context (CpG, CHG and CHH) were determined according to different criteria: 1) for CpG, the number of CpG in each window was ≥ 5, the absolute value of the difference in methylation ratio was ≥ 0.2, and *q* ≤ 0.05; 2) for CHG, the number in a window was ≥ 5, the absolute value of the difference in methylation ratio was ≥ 0.2, and *q* ≤ 0.05; 3) for CHH, the number in a window was ≥ 5, the absolute value of the difference in methylation ratio was ≥ 0.15, and *q* ≤ 0.05; 4) for all C, the number in a window was ≥ 5, the absolute value of the difference in methylation ratio was ≥ 0.2, and *q* ≤ 0.05.

### Lactate content determination

To determine whether there is a difference in the lactate content in the intestines of the resistant and susceptible families, we determined the lactate levels in their intestine samples using ion exchange column liquid chromatography with HPLC. The experiment was conducted using Agilent C_18_ AQ column, which utilizes a C18 stationary phase of 250 × 4.6 mm id and a particle size of 5 μm. The mobile phases were 0.1% phosphoric acid-water and methanol.

### Lactate feeding to shrimp to detect resistance to *VP*_*AHPND*_

To test the functions of lactate in disease resistance, we fed lactate to shrimp to assess resistance to *VP*_*AHPND*_. A basal diet was purchased from Xiamen Hailin Technology Co., Ltd. Different quantities of lactate mixed with a small amount of egg white were added to the basal diet. The weights of the basal diet, egg white and lactate follows ratios of 119: 5: X, where X = 0, 1, and 10. A total of 108 healthy shrimp (body weight = 8.87 ± 1.94 g and body length = 8.91 ± 0.78 cm) were equally divided into three groups (shrimp groups that were fed with three different concentrations of lactate), and each group had three replications. The shrimp were fed three times a day and lasted for five days. Then, each shrimp group was challenged with *VP*_*AHPND*_ to test their tolerance to pathogen infection. Based on the pre-experiment results, a dose of 3.0 × 10^6^ CFU/mL was used for further immersion infection. Bacterial suspension was added directly to seawater in the experimental tanks to obtain an approximate bacterial density of 3.0 × 10^6^ CFU/mL. The mortality rates were recorded at 12 h and 24 h after immersion infection.

### RNA interference (RNAi) of *LDH* in shrimp

In order to identify the functions of *LDH* in disease resistance, we performed RNAi experiments on *LDH* in shrimp. Double-stranded RNA (dsRNA) of *LDH* with an insert length of 639 bp was designed and synthesized using a transcriptAid T7 High Yield Transcription Kit (K0441, Thermo Fisher Scientific, Beijing, China). The primers used for full-length amplification, RT-qPCR, and RNAi were designed based on the *LDH* cDNA sequences (Table C in S1 Text). Transcription templates were prepared by PCR using gene-specific primers with a T7 polymerase promoter sequence. The dsRNA of the enhanced green fluorescent protein (EGFP) gene was also synthesized. The dsEGFP group (injected with EGFP dsRNA) and PBS (solvent) group were used as negative controls. The concentration and quality of synthesized dsRNA were detected using a spectrophotometer (Thermo Fisher Scientific, MA, USA) and 1.5% (w/v) agarose gel electrophoresis.

Shrimp were cultured in the seawater at 25 ± 1°C with the salinity of 30 ppt for 3 days prior to the experiments. The optimum interference concentration of ds-LDH was determined by a pre-experiment. After that, a four-day RNAi experiment was performed in shrimp. A total of 189 healthy shrimp (body weight = 9.21 ± 2.30 g and body length = 8.92 ± 1.11 cm) were equally divided into three groups (the PBS group, ds-EGFP group, and ds-LDH group; each group was divided into three replications) and injected with 10 μL of PBS, 10 μL of ds-EGFP (0.2 μg/μL), and 10 μL of ds-LDH (0.2 μg/μL), respectively. Shrimp were injected twice, every two days. After four days, the shrimp from each group were challenged with *VP*_*AHPND*_ to test their tolerance to pathogen infection. Based on the pre-experiment results, a dose of 1.0 × 10^6^ CFU/mL was used for further immersion infection. The mortality rates were recorded at 0 h, 12 h, 18 h, and 24 h after immersion infection.

### Methylation inhibitor 5-azacytidine treatment experiment

To examine whether methylation changes can regulate intestinal microbial community structure, we inhibited DNA methylation using 5-azacytidine (5-Azac, Sigma-Aldrich) and conducted microbiome sequencing and analysis on treated samples. The optimum inhibition concentration of 5-Azac was determined by a pre-experiment. Then, a five-day 5-Azac (0.5 mg/L) treatment experiment was performed on laboratory-cultured shrimp (body weight of 2.05 ± 0.41 g). Preinjection samples and PBS-injected samples were used as the control groups. Hepatopancreas and intestine samples were collected from 15 individuals per group (5-Azac, Control, and PBS).

The expression profiles of three selected genes (*DMNT2*, *LDH*, and *PDIA3*) in the hepatopancreas were examined by real-time qPCR (RT-qPCR). To investigate whether the intestinal microbial community was affected by 5-Azac, the intestinal samples were subjected to microbiome sequencing and analysis according to the methods described above. Samples from three individuals were mixed into a single sample in microbiome sequencing.

## Supporting information

S1 Text This PDF file contains Figs A–O and Tables A-C. Fig A. Survive rate of the 87 full-sib families of *L. vannamei* after Vibrio infection. Fig B. Microbial community structure in resistant and susceptible families at genus level. “*” indicates significant difference of the content of Shewanella between resistant and susceptible families. Fig C. Venn plot of DEGs in the hepatopancreas and intestine tissues of the two shrimp families. Fig D. PCA analysis of gene expression profiles in the four groups of hepatopancreas samples. Fig E. KEGG enrichment of the genes with genetic mutations. Fig F. A schematic representation of the DNA methylation patterns in the *L. vannamei* genome. Track 1 (RHP0): DNA methylation profiles of RHP0. Track 2 (RHP6): DNA methylation profiles of RHP6. Track 3 (SHP0): DNA methylation profiles of SHP0. Track 4 (SHP6): DNA methylation profiles of SHP6. Track 5 (DMR(RHP0-RHP6)): DMRs of the comparison between RHP0 and RHP6. Track 6 (DMR(RHP0-SHP0)): DMRs of the comparison between RHP0 and SHP0. Track 7 (Complete methylation sites): all the complete methylation sites (methylation level > 90%) in the genome. Fig G. CpG methylation clustering analysis of all the sequencing samples. Fig H. Methylation profile along gene body in the samples of susceptible (SHP6) and resistant (RHP6) families post V. parahaemolyticus infection. Fig I. DNA methylation levels in various genomic regions. (A) The methylation level of total methylated cysteine. (B) The methylation level of CpG. (C) The methylation level of CHG. (D) The methylation level of CHH. Fig J. DNA methylation profile of a representative gene (LVAN07826). The analyzed region stems from the upstream 2 kb to the downstream 2 kb. The dark blue blocks indicate exons of the gene, and the yellow blocks indicate the DMRs. Fig K. The Spearman correlation of the DNA methylation level and gene expression level in the regions of gene body and upstream and downstream 2 kb regions. Fig L. The methylation levels of the genes with various expression level. According to the expression levels, genes were classified into four groups: none expression (FPKM ≤ 1), low expression level (1 < FPKM ≤ 10), middle expression level (10 < FPKM ≤ 100), high expression level (FPKM > 100). TSS indicates transcription start site and TTS indicates transcription stop site. Genes with higher expression level have lower methylation level. Fig M. DMC summary for each sequence context (total mC, CG, CHG and CHH) in various comparison groups. Fig N. Heatmap of the gene expression and DNA methylation profiles of the interactions of DEGs and DMGs. Fig O. The significance difference test of the abundance of three most abundant genus. “*” indicates significant difference (the Tukey HSD test, p < 0.05) of the abundance of Shewanella between the group of 5-Azac and the groups of Control and PBS. Table A. The significance difference test of the contents of four most abundant genus. Table B. Summary of methylome sequencing data. Table C. The primers used in this paper. The forward and reverse primers are represented by F and R, respectively.
(PDF)

S1 Data Summary of SNPs that identified in the RNA-seq data. S1 Data contain SNPs that identified in the RNA-seq data.
(XLSX)
