# Supplementary material for: Shrimp shapes a resistance trait against vibriosis by memorizing the colonization resistance of intestinal microbiota
Source: PLoS Pathog. 2024 Jul 11;20(7):e1012321. doi: 10.1371/journal.ppat.1012321 (PMC11239079; doi:10.1371/journal.ppat.1012321)
Supplement: S1 Text — This PDF file contains Figs A–O and Tables A-C. Fig A. Survive rate of the 87 full-sib families of L. vannamei after Vibrio infection. Fig B. Microbial community structure in resistant and susceptible families at genus level. “*” indicates significant difference of the content of Shewanella between resistant and susceptible families. Fig C. Venn plot of DEGs in the hepatopancreas and intestine tissues of the two shrimp families. Fig D. PCA analysis of gene expression profiles in the four groups of hepatopancreas samples. Fig E. KEGG enrichment of the genes with genetic mutations. Fig F. A schematic representation of the DNA methylation patterns in the L. vannamei genome. Track 1 (RHP0): DNA methylation profiles of RHP0. Track 2 (RHP6): DNA methylation profiles of RHP6. Track 3 (SHP0): DNA methylation profiles of SHP0. Track 4 (SHP6): DNA methylation profiles of SHP6. Track 5 (DMR(RHP0-RHP6)): DMRs of the comparison between RHP0 and RHP6. Track 6 (DMR(RHP0-SHP0)): DMRs of the comparison between RHP0 and SHP0. Track 7 (Complete methylation sites): all the complete methylation sites (methylation level > 90%) in the genome. Fig G. CpG methylation clustering analysis of all the sequencing samples. Fig H. Methylation profile along gene body in the samples of susceptible (SHP6) and resistant (RHP6) families post V. parahaemolyticus infection. Fig I. DNA methylation levels in various genomic regions. (A) The methylation level of total methylated cysteine. (B) The methylation level of CpG. (C) The methylation level of CHG. (D) The methylation level of CHH. Fig J. DNA methylation profile of a representative gene (LVAN07826). The analyzed region stems from the upstream 2 kb to the downstream 2 kb. The dark blue blocks indicate exons of the gene, and the yellow blocks indicate the DMRs. Fig K. The Spearman correlation of the DNA methylation level and gene expression level in the regions of gene body and upstream and downstream 2 kb regions. Fig L. The methylation levels of the [file ppat.1012321.s001.pdf]

Shrimp shapes a resistance trait against vibriosis by memorizing the colonization resistance of intestinal microbiota

Short title: Shrimp memorizes colonization resistance

Authors

Jianbo Yuan[1,2] , Yang Yu[1,2], Shihao Li[1,2], Xiaojun Zhang[1,2] , Chuntao Zhang[1,3], Roujing Li[1], Jie Hu[1], Shuqing Si[1,3], Chengyi Zhang[1,3], Jianhai Xiang[1,2], Fuhua Li[1,2,*]

Affiliations

[1] CAS and Shandong Province Key Laboratory of Experimental Marine Biology, Center for Ocean Mega-Science, Institute of Oceanology, Chinese Academy of Sciences, Qingdao, China.

[2] Key Laboratory of Breeding Biotechnology and Sustainable Aquaculture, Chinese Academy of Sciences, Wuhan, China.

[3] University of Chinese Academy of Sciences, Beijing, China

[*] Correspondence and requests for materials should be addressed to F.L. (fhli@qdio.ac.cn, Orcid ID: 0000-0001-8693-600X).

Figures

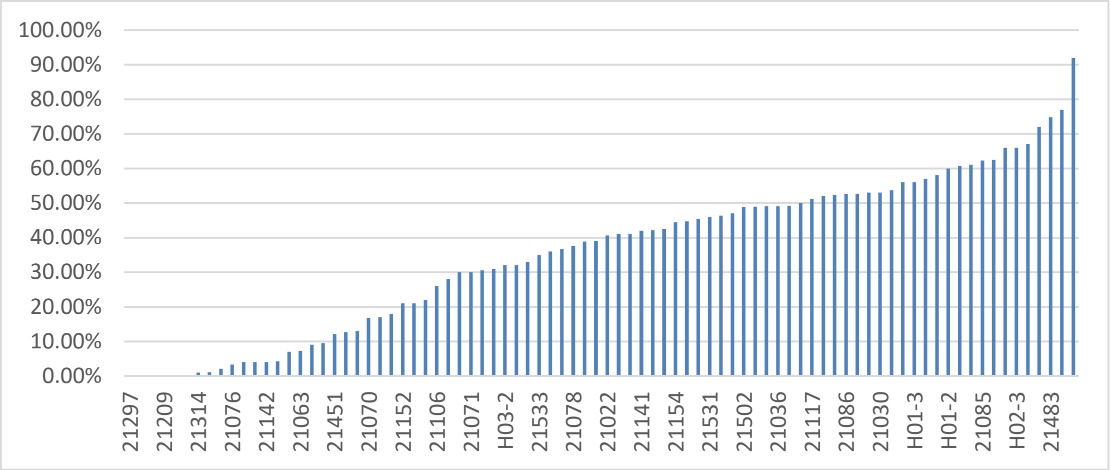

Fig A. Survive rate of the 87 full-sib families of *L. vannamei* after *Vibrio* infection.

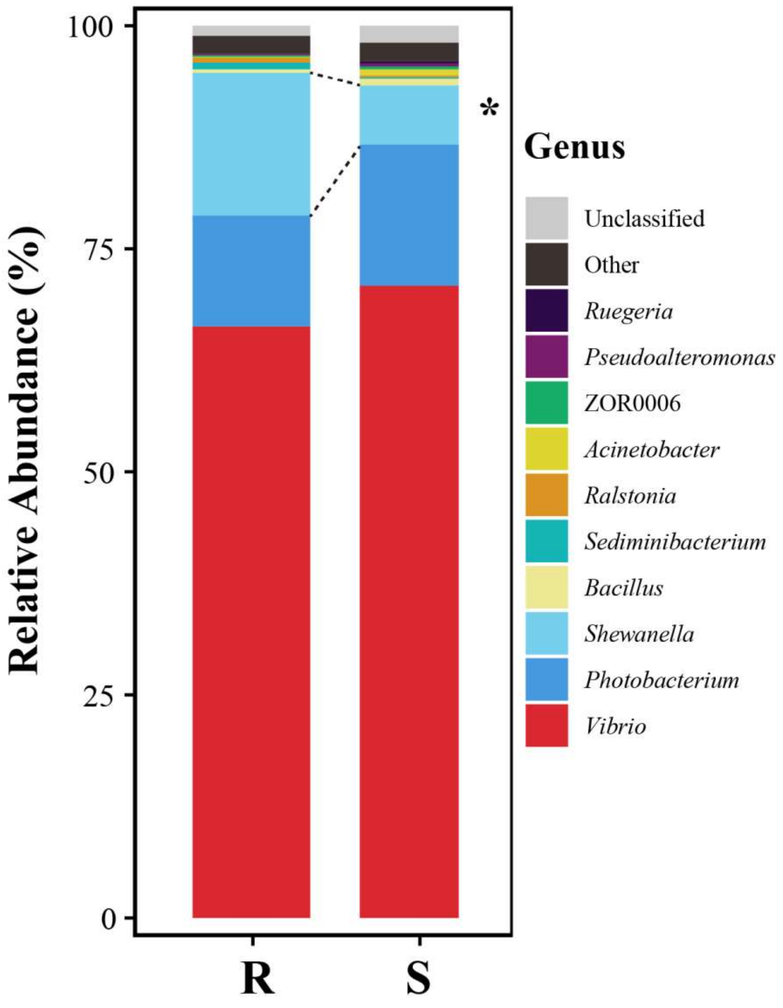

Fig B. Microbial community structure in resistant and susceptible families at genus level. “*” indicates significant difference of the content of *Shewanella* between resistant and susceptible families.

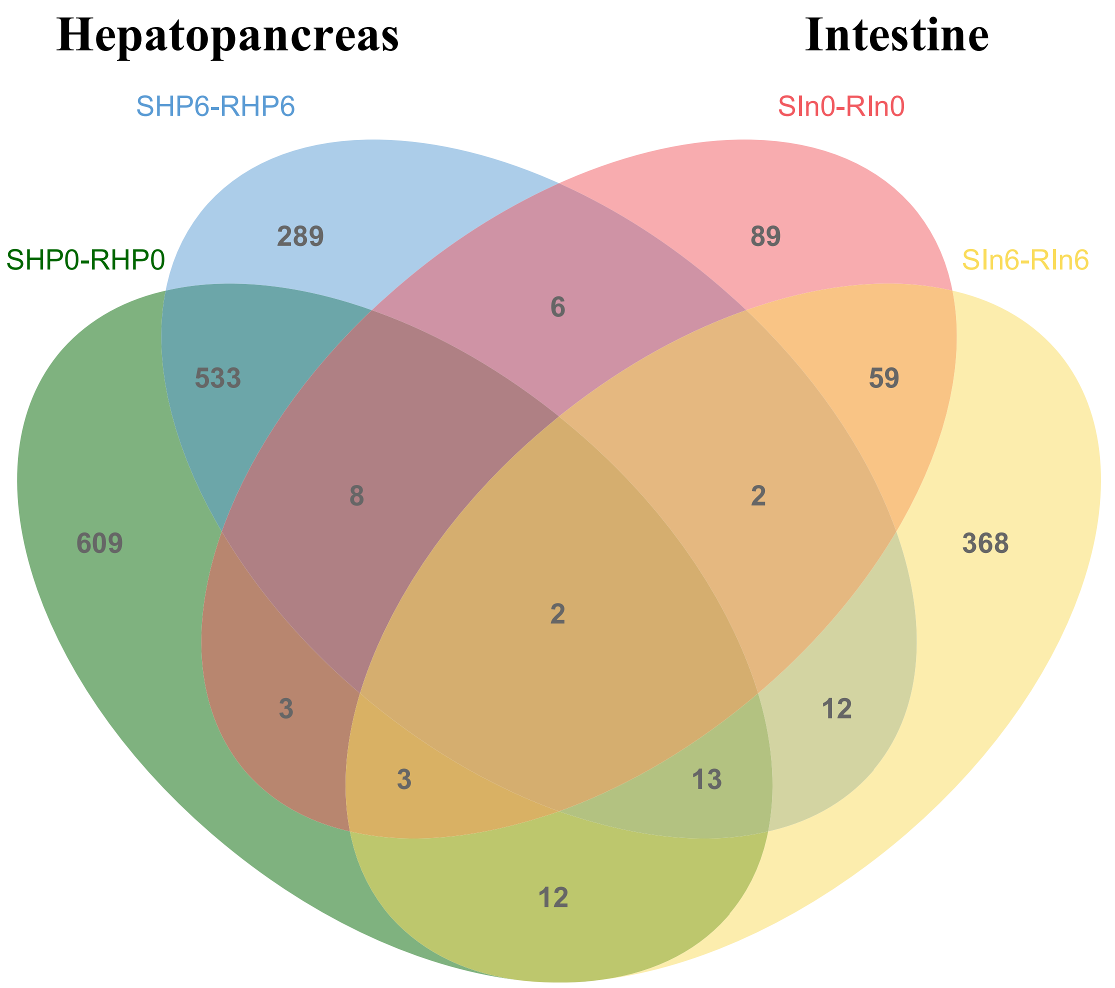

Fig C. Venn plot of DEGs in the hepatopancreas and intestine tissues of the two shrimp families.

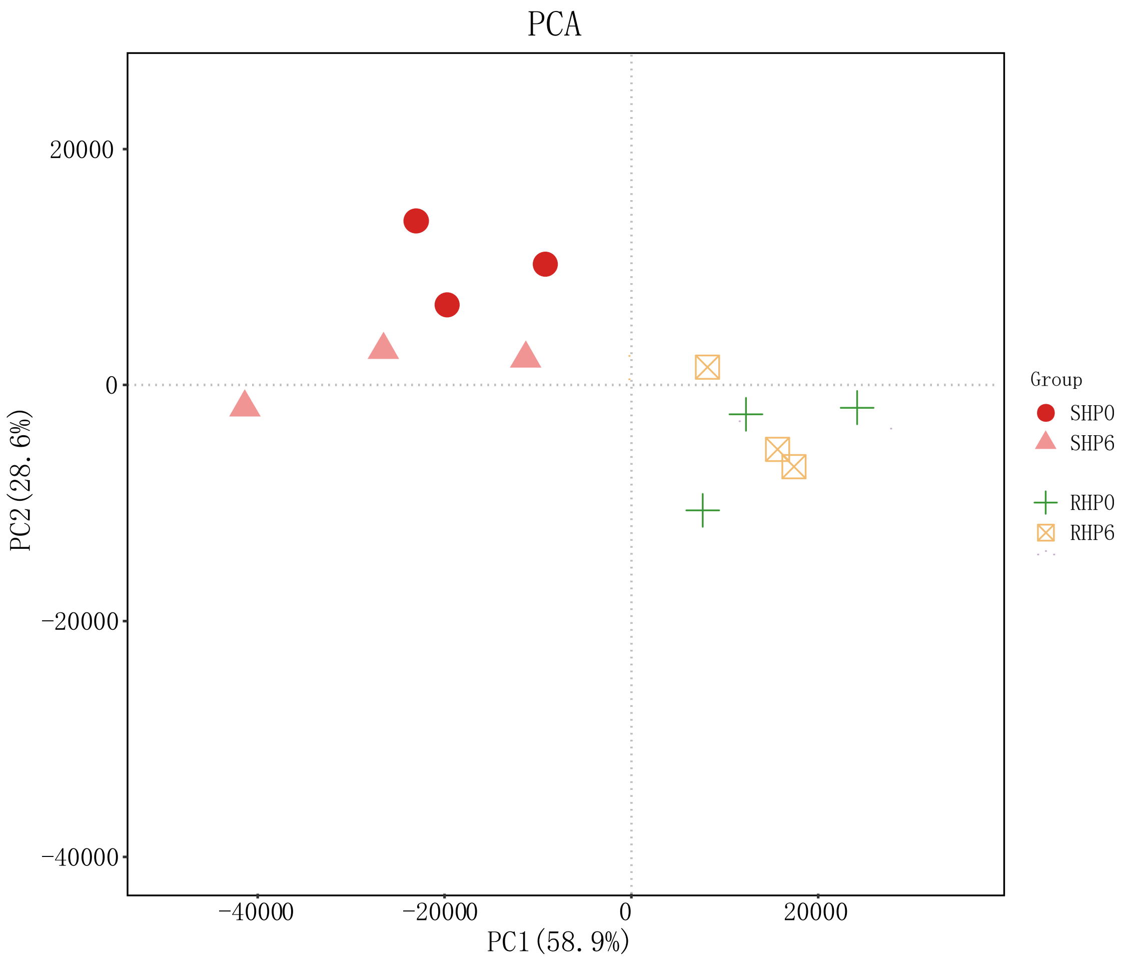

Fig D. PCA analysis of gene expression profiles in the four groups of hepatopancreas samples.

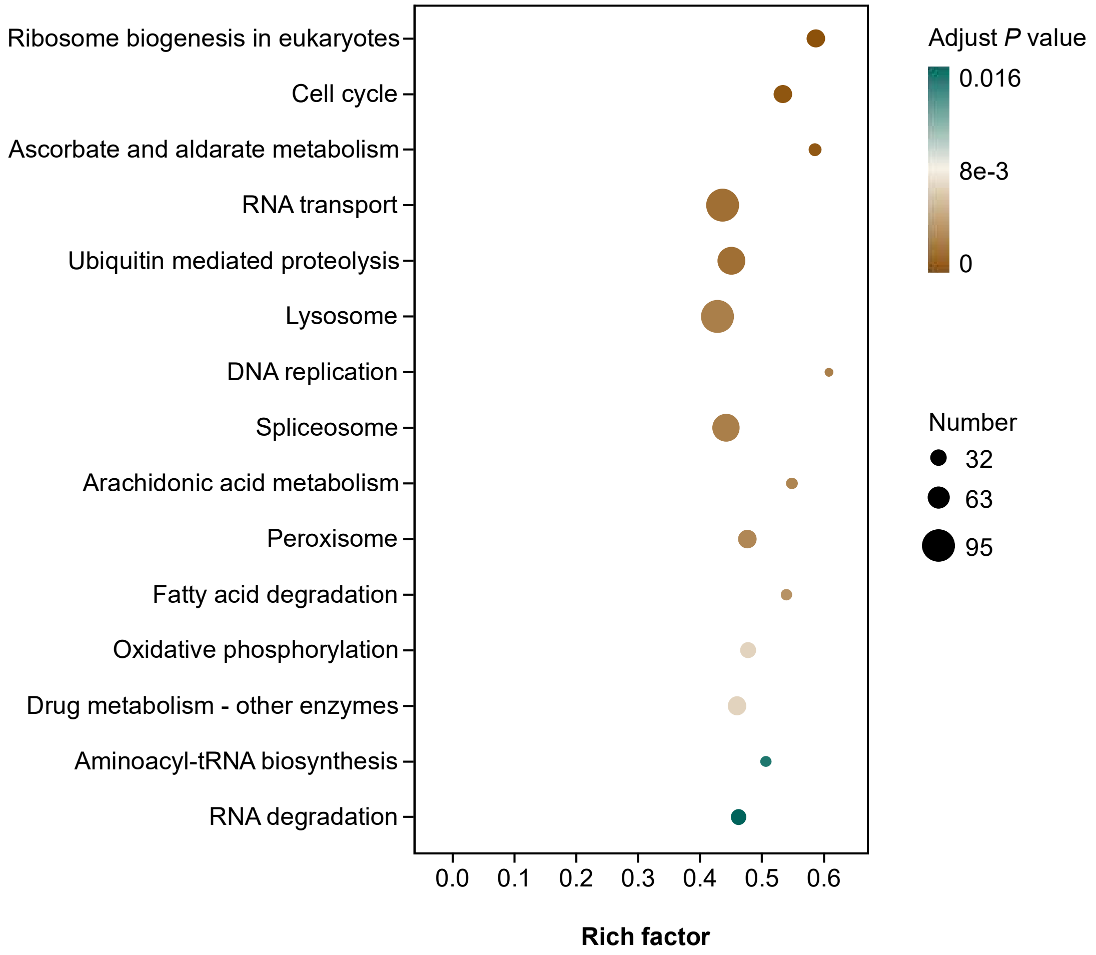

Fig E. KEGG enrichment of the genes with genetic mutations.

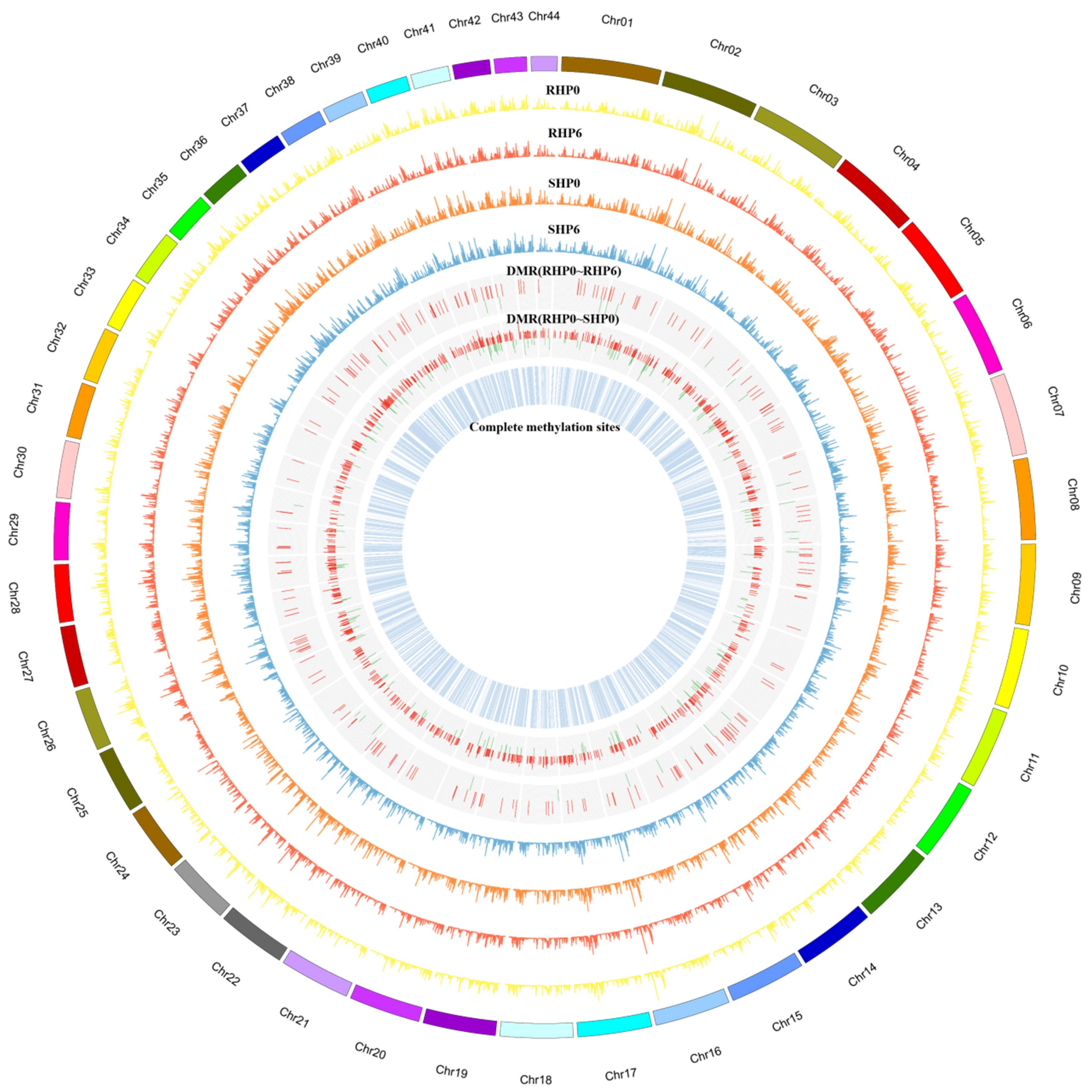

Fig F. A schematic representation of the DNA methylation patterns in the *L. vannamei* genome. Track 1 (RHP0): DNA methylation profiles of RHP0. Track 2 (RHP6): DNA methylation profiles of RHP6. Track 3 (SHP0): DNA methylation profiles of SHP0. Track 4 (SHP6): DNA methylation profiles of SHP6. Track 5 (DMR(RHP0-RHP6)): DMRs of the comparison between RHP0 and RHP6. Track 6 (DMR(RHP0-SHP0)): DMRs of the comparison between RHP0 and SHP0. Track 7 (Complete methylation sites): all the complete methylation sites (methylation level > 90%) in the genome.

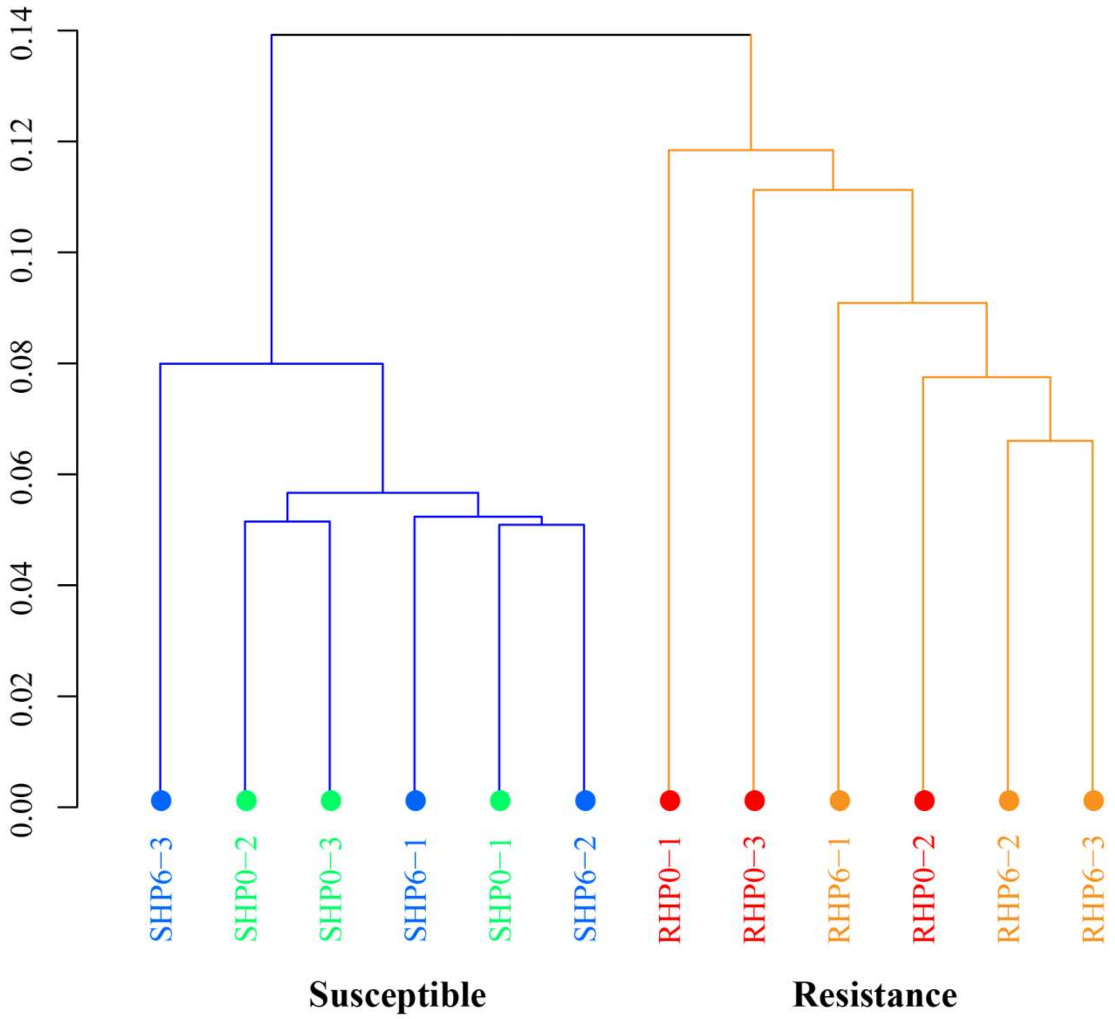

Fig G. CpG methylation clustering analysis of all the sequencing samples.

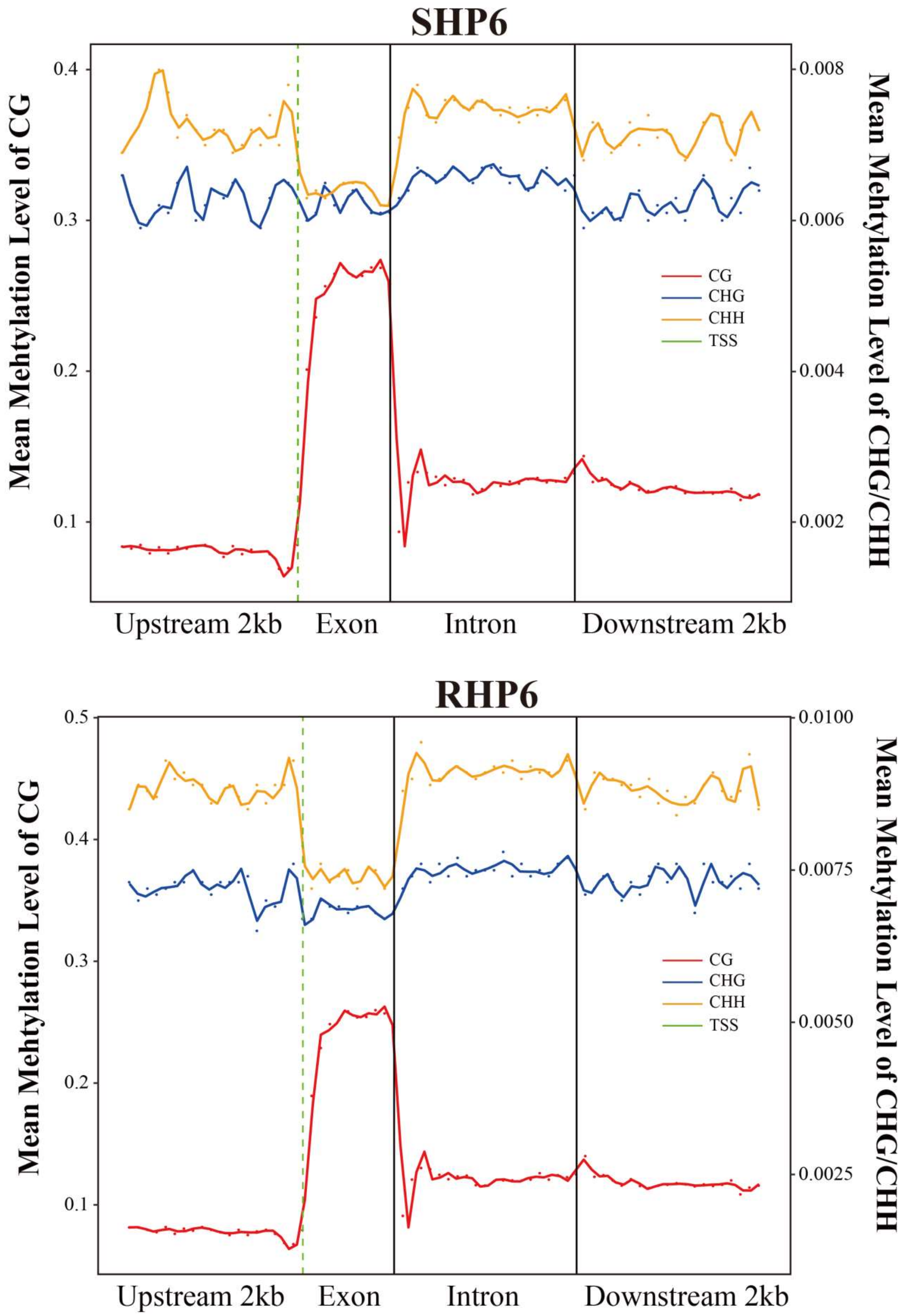

Fig H. Methylation profile along gene body in the samples of susceptible (SHP6) and resistant (RHP6) families post *V. parahaemolyticus* infection.

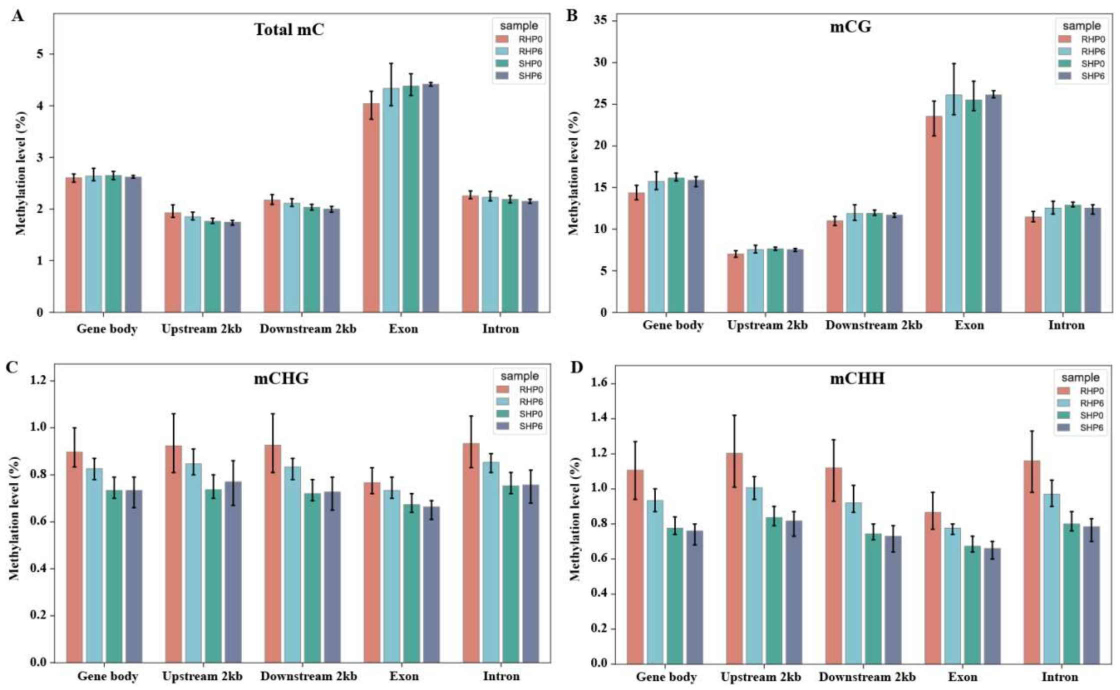

Fig I. DNA methylation levels in various genomic regions. (A) The methylation level of total methylated cysteine. (B) The methylation level of CpG. (C) The methylation level of CHG. (D) The methylation level of CHH.

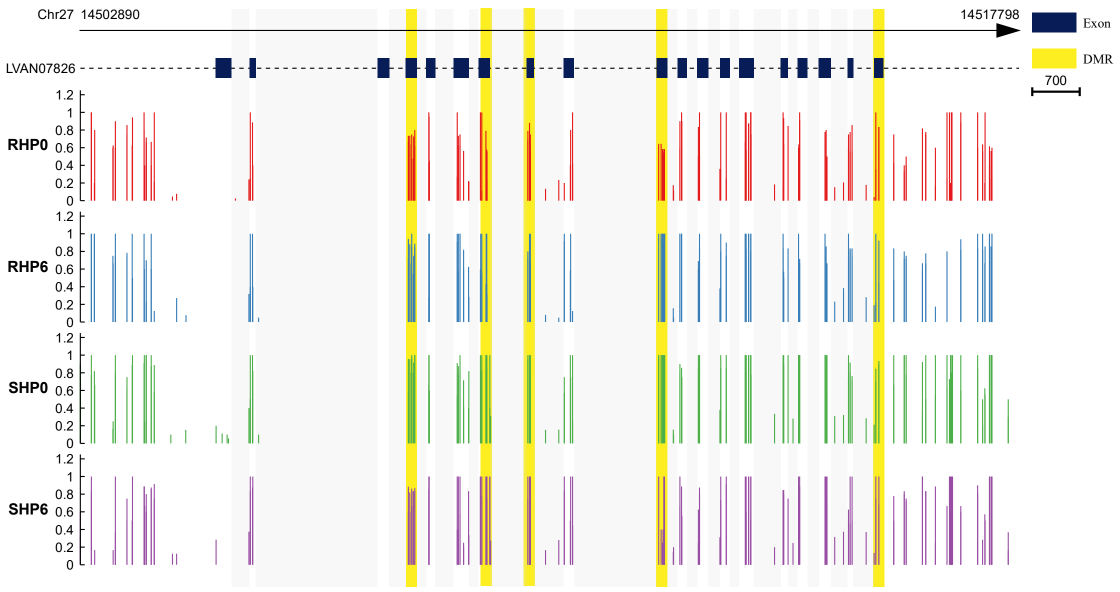

Fig J. DNA methylation profile of a representative gene (LVAN07826). The analyzed region stems from the upstream 2 kb to the downstream 2 kb. The dark blue blocks indicate exons of the gene, and the yellow blocks indicate the DMRs.

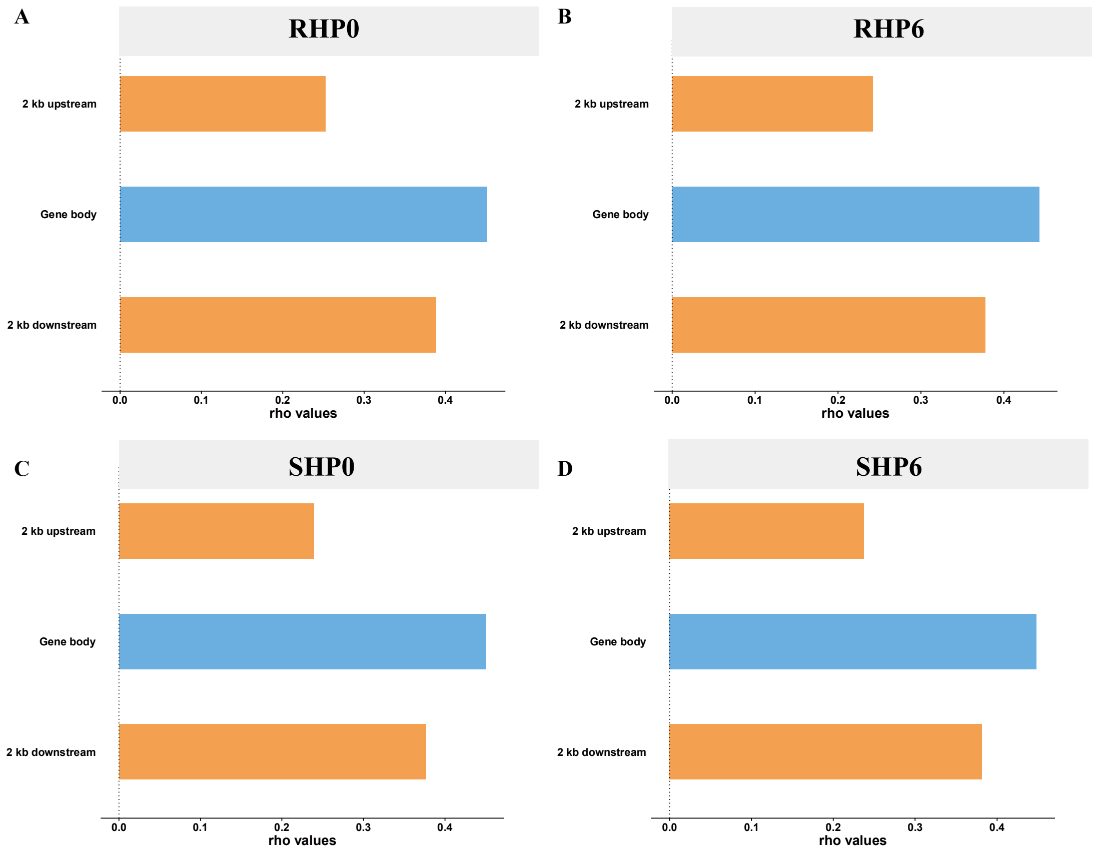

Fig K. The Spearman correlation of the DNA methylation level and gene expression level in the regions of gene body and upstream and downstream 2 kb regions.

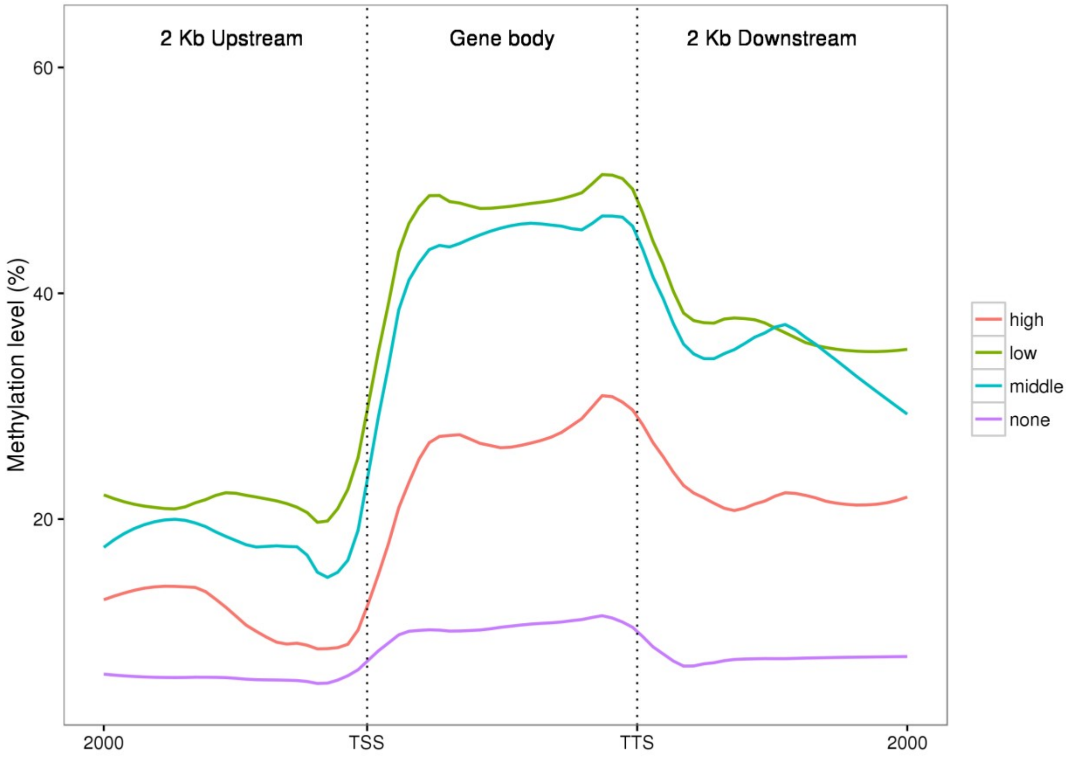

Fig L. The methylation levels of the genes with various expression level. According to the expression levels, genes were classified into four groups: none expression (FPKM ≤ 1), low expression level (1 < FPKM ≤ 10), middle expression level (10 < FPKM ≤ 100), high expression level (FPKM > 100). TSS indicates transcription start site and TTS indicates transcription stop site. Genes with higher expression level have lower methylation level.

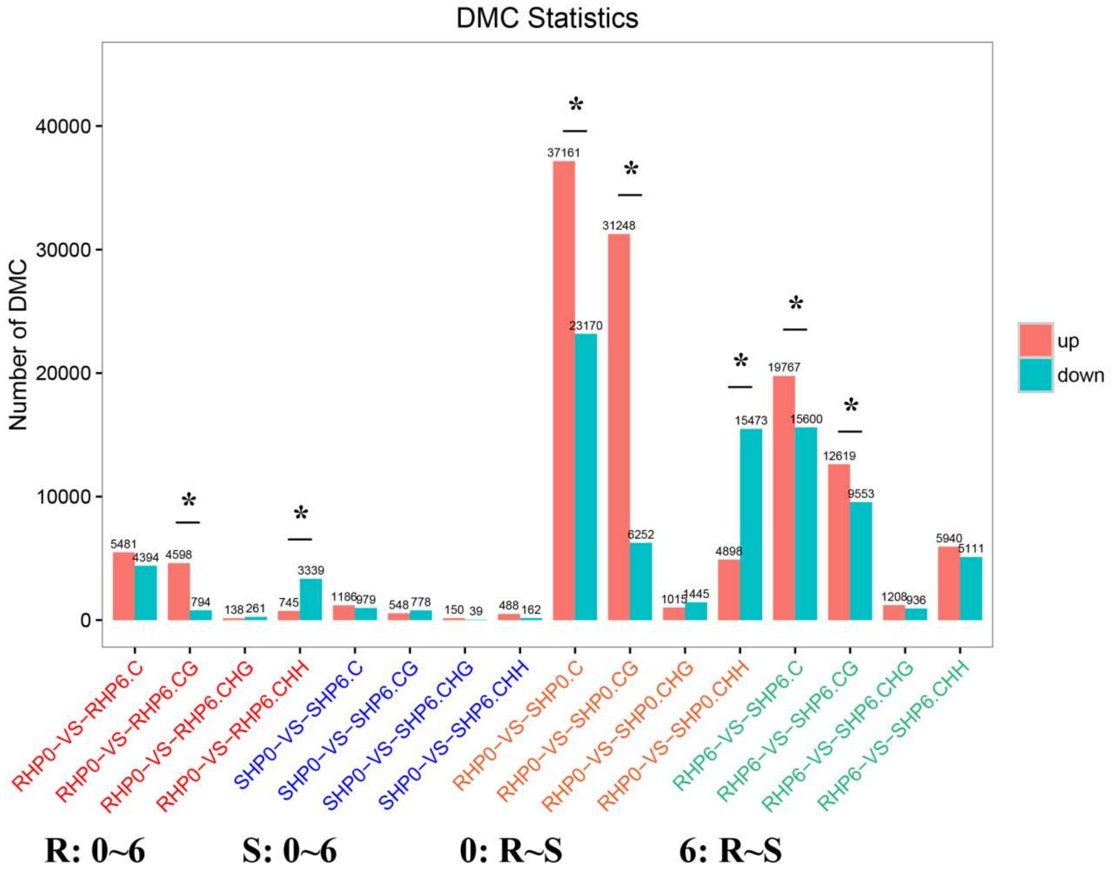

Fig M. DMC summary for each sequence context (total mC, CG, CHG and CHH) in various comparison groups.

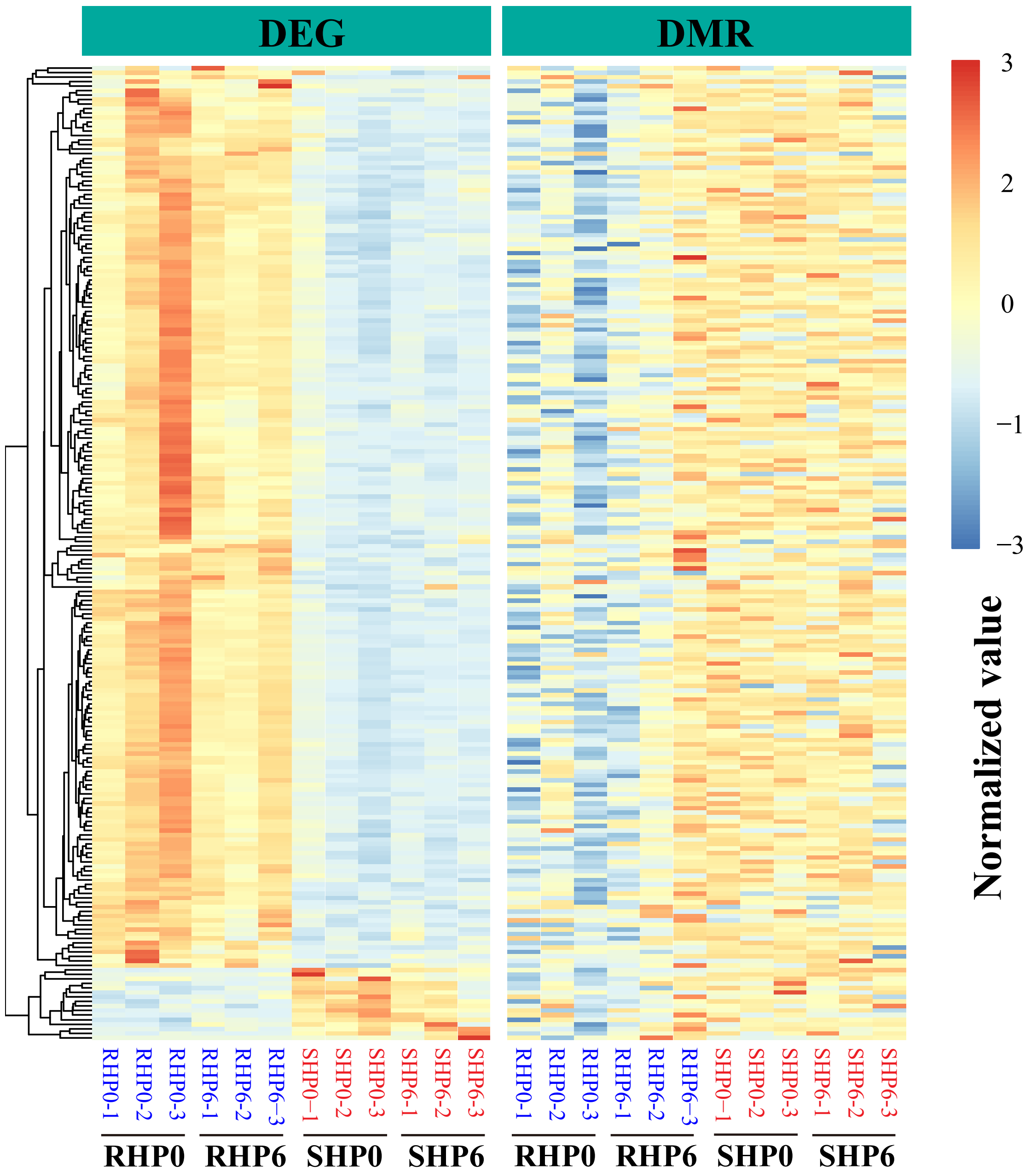

Fig N. Heatmap of the gene expression and DNA methylation profiles of the interactions of DEGs and DMGs.

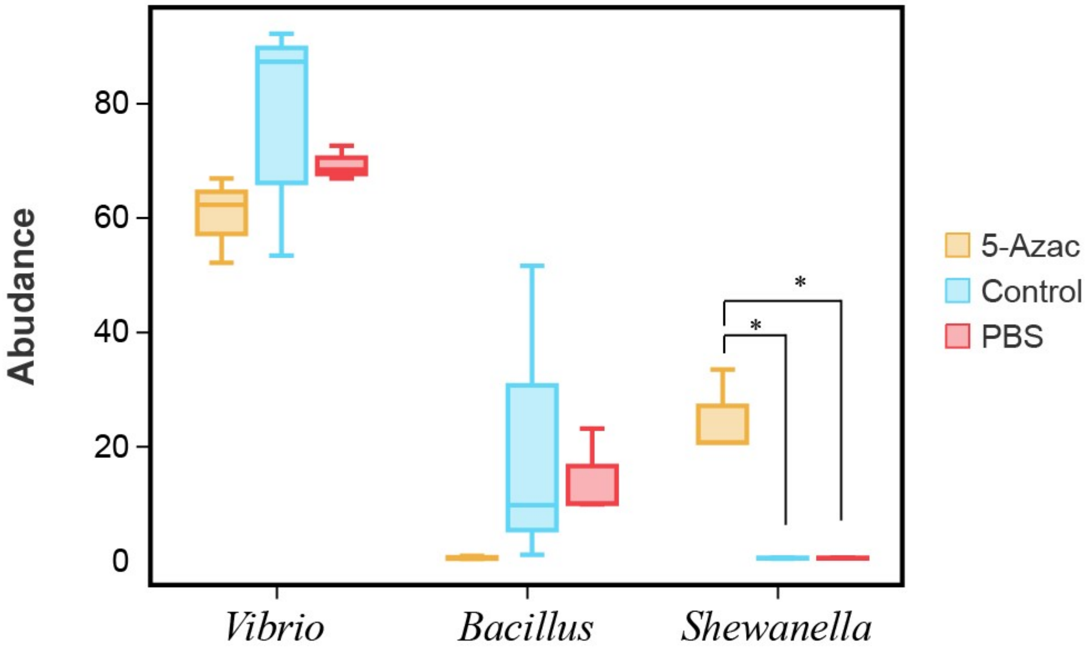

Fig O. The significance difference test of the abundance of three most abundant genus. “*” indicates significant difference (the Tukey HSD test, $p < 0.05$) of the abundance of *Shewanella* between the group of 5-Azac and the groups of Control and PBS.

Tables

Table A. The significance difference test of the contents of four most abundant genus.

Genus	S (%)	R (%)	fold(R/S)	p-value	Significant
Vibrio	70.8631	66.31203	0.935777	0.453917	no
Photobacterium	15.811	12.42216	0.785666	0.471506	no
Shewanella	6.650807	15.99774	2.405383	0.037492	yes
Bacillus	0.751167	0.37138	0.494404	0.537787	no

Table B. Summary of methylome sequencing data.

Samples	Total Reads	Sequence Depth	Mapped Reads	Mapped Ratio(%)
RHP0-1	4.11E+08	46.24346	1.34E+08	32.68
RHP0-2	3.52E+08	39.72374	1.2E+08	34
RHP0-3	3.4E+08	37.5664	1.15E+08	33.71
RHP6-1	3.43E+08	38.00251	1.21E+08	35.2
RHP6-2	3.62E+08	40.54041	1.35E+08	37.47
RHP6-3	3.41E+08	38.83951	1.16E+08	34
SHP0-1	3.54E+08	40.1067	1.31E+08	37
SHP0-2	3.31E+08	36.84229	1.32E+08	39.97
SHP0-3	3.3E+08	36.97494	1.27E+08	38.64
SHP6-1	3.28E+08	37.05202	1.24E+08	37.79
SHP6-2	3.27E+08	36.9792	1.24E+08	37.92
SHP6-3	3.19E+08	36.62132	1.08E+08	33.89

Table C. The primers used in this paper. The forward and reverse primers are represented by F and R, respectively.

**Full-length primer**	
LDH_FL_F	GAAGATTGATGCAAACACCGA
LDH_FL_R	TGGTATCGACAAGGATGTGTT
**Quantitative primer**	
LDH_Q_F	GAGAGTCTCGTTTGTCCCTT
LDH_Q_R	CAGTTTCCATGCAACGTAGG
18S_Q_F	TATACGCTAGTGGAGCTGGAA
18S_Q_R	GGGGAGGTAGTGACGAAAAAT
**RNAi primer**	
ds-LDH_F	TAATACGACTCACTATAGGGGAAGATTGATGCAAACACCGA
ds-LDH_R	TAATACGACTCACTATAGGGAACACATCCTTGTCGATACCA
